# USP11 acts as a histone deubiquitinase functioning in chromatin reorganization during DNA repair

**DOI:** 10.1093/nar/gkz726

**Published:** 2019-08-28

**Authors:** Xia Ting, Lu Xia, Jianguo Yang, Lin He, Wenzhe Si, Yongfeng Shang, Luyang Sun

**Affiliations:** 1 Key Laboratory of Carcinogenesis and Translational Research (Ministry of Education), Department of Biochemistry and Molecular Biology, School of Basic Medical Sciences, Peking University Health Science Center, Beijing 100191, China; 2 Department of Integration of Chinese and Western Medicine, School of Basic Medical Sciences, Peking University Health Science Center, Beijing 100191, China; 3 Department of Biochemistry and Molecular Biology, School of Basic Medical Sciences, Capital Medical University, Beijing 100069, China; 4 Department of Laboratory Medicine, Peking University Third Hospital, Beijing 100191, China

## Abstract

How chromatin dynamics is regulated to ensure efficient DNA repair remains to be understood. Here, we report that the ubiquitin-specific protease USP11 acts as a histone deubiquitinase to catalyze H2AK119 and H2BK120 deubiquitination. We showed that USP11 is physically associated with the chromatin remodeling NuRD complex and functionally involved in DNA repair process. We demonstrated that USP11-mediated histone deubiquitination and NuRD-associated histone deacetylation coordinate to allow timely termination of DNA repair and reorganization of the chromatin structure. As such, USP11 is involved in chromatin condensation, genomic stability, and cell survival. Together, these observations indicate that USP11 is a chromatin modifier critically involved in DNA damage response and the maintenance of genomic stability.

## INTRODUCTION

Eukaryotic cells evolve highly efficient DNA repair system to guide the integrity of the genome in response to a plethora of exogenous as well as endogenous DNA insults. Unrepaired or misrepaired DNA damages can cause gross chromosome rearrangements or mutations at break sites, eventually leading to tumorigenesis, inflammatory diseases, and ageing (1). DNA double-strand breaks (DSBs), which are exceedingly dangerous chromosomal lesions as they entail physical cleavage of the DNA backbone, trigger two mechanistically distinct pathways of DNA damage response (DDR), non-homologous end-joining (NHEJ) and homologous recombination (HR) (2–4). Both the initial response at DSB sites and subsequent spreading of the DNA damage alarms involve extensive dynamic post-translational modifications (PTMs) of histones and non-histones, including phosphorylation and ubiquitylation (5–7).

Ubiquitination, the covalent attachment of ubiquitin (Ub) to target proteins, involves sequential enzymatic reactions mediated by E1, E2 and E3 enzymes (8). Monoubiquitination or polyubiquitination with different linkages in target proteins might play different functional roles during the DDR, including recruitment of DDR-dedicated proteins, modulation of protein activity, and targeting proteins for degradation by the 26S proteasome (8–10). Ubiquitination can be reversed by Ub proteases called deubiquitinating enzymes (DUBs). The human genome encodes ∼90 putative DUBs that can be classed into five distinct families based on their catalytic domains, with the ubiquitin-specific protease (USP) subclass represents the bulk of the DUBs (11). Biologically, several DUBs have been implicated in DNA damage response. For example, USP24 is reported to act as a deubiquitinase of p53 to mediate UV damage response (12); BRCC36 specifically hydrolyses Ub-K63 polymers to regulate 53BP1 accumulation in cell responses to DSBs (13); and OTUB1 antagonizes RNF168-dependent DSB signaling by binding to and non-canonically inhibiting UBC13, the cognate E2 enzyme for RNF168 (14). In fact, a systematic screening of DUB for their roles in the maintenance of genome integrity indicates that as many as 23 DUBs are potentially involved in DSB repair and the G_2_/M checkpoint (15). Ultimately, the multitude and magnitude of DUBs impacting on DNA damage response need experimental validation, and the substrates and exact functions of these DUBs in the maintenance of genome stability need further elucidation.

Histone modifications including ubiquitination influence gene transcription, DNA damage repair, and development by remodeling the chromatin structure. In cellular response to DSBs, highly dynamic PTMs of histones are crucial for DNA damage recognition and/or signaling, repair of DNA lesions, and release of cell-cycle arrest. Among these PTMs, ATM-dependent phosphorylation and E3 ligase-mediated ubiquitination form an integral part of the regulatory network to guide the DNA damage response (5,9,16–18). Specifically, RNF168-catalyzed H2AK15ub extended by RNF8 to form K63-linked ubiquitin chain is important for the recruitment of 53BP1 and BRCA1 (19–21), while BRCA1 dimerizes with BARD1 and ubiquitinates H2AK127/129 to promote HR repair (22). H2BK120 monoubiquitination by RNF20/RNF40 is crucial for damage checkpoint activation and timely initiation of both HR and NHEJ repair (23,24), whereas BAP1 deubiquitinates H2AK119ub to mediate DSB repair via HR pathway (25). In addition, USP51 deubiquitinates H2AK13/15 to alter RAP80 and 53BP1 localization (26), while USP16 deubiquitinase H2AK119 and H2AK15 to finely tune cellular responses to DNA damage and local transcriptional restoration after recovery from DDR (27). Moreover, USP22-associated SAGA complex promotes H2BK120 deubiquitination to regulate DNA repair and class switch recombination at the immunoglobulin locus (28). Given the importance of histone ubiquitination in recognition of repair proteins and chromatin reorganization at damaged sites, it is equally important to clear histone ubiquitinations after repair for chromatin restoration (6,29). However, how histone ubiquitination is reversed in a timely manner to mediate recovery from DNA damages and acts in coordination with other PTMs such as histone acetylation for chromatin remodeling remain to be understood.

USP11 is a member of USP family protein that has been reported to act as a deubiquitinase targeting HPV-16E7, PML, p21, E2F1 and XIAP and implicated in the initiation and progression of several malignancies including cervical, brain, lung, liver and colon cancer (30–38). Recently, a functional genomic screen showed that USP11 inhibits cell growth and survival by repressing ERα transcriptional activity. Tissue microarray together with public database analysis showed a significant correlation between high USP11 expression and poor prognosis in ER^+^ patients, supporting USP11 as a novel therapeutic target for breast cancer (39). In addition, a systematic screen by neutral comet assays indicates that USP11 is potentially involved in DSB repair (15), and it has also been reported that USP11 acts as a DUB for BRCA2 and PALB2, facilitating the formation of a stable BRCA1-PALB2-BRCA2 complex and inducing homologous recombination (HR) repair in G_1_ cells (40,41). Furthermore, a screen with siRNA library showed that USP11 silencing sensitizes cells to a PARP inhibitor, accompanied by a spontaneous activation of DNA double-strand break repair (42). Finally, USP11 can also target γH2AX for deubiquitination to modulate the recruitment of 53BP1 and ubiquitin-conjugated proteins to DSB sites (43) and target XPC for deubiquitination to regulate nucleotide excision repair (NER) (44). However, whether USP11 could target histone H2A and H2B is currently unknown.

In the current study, we report that USP11 is a deubiquitinase for H2AK119 and H2BK120. We showed that USP11 is physically associated with the NuRD complex and functionally linked to efficient DNA repair. We demonstrated that USP11 protects cells from genotoxic insults and is required for chromatin condensation, genomic stability, and cell survival.

## MATERIALS AND METHODS

### Plasmids

The cDNA for wild-type USP11, H2A, H2B or HDAC2 was amplified by PCR and ligated into pcDNA3.1(–) plasmid containing FLAG tag. USP11/C318S, H2AK13/15R, H2AK118/119R and H2BK120R were generated by using QuikChange Lightning Site-Directed Mutagenesis Kit. GFP-USP11, GFP-MTA2, GFP-HDAC2 were constructed by cloning full-length of USP11, MTA2, HDAC2 into pEGFP-N1 vector.

### Antibodies and reagents

Antibodies used: anti-FLAG (F3165), anti-β-actin (A1978), anti-HDAC2 (H3159) from Sigma; anti-USP11 (ab109232), anti-H3 (ab1791), anti-PAR (ab14459), anti-MTA2 (ab50209), anti-GST (ab19256), anti-RNF20 (ab181032) from Abcam; anti-Mi-2β (sc-365638), anti-MTA2 (sc-55566), anti-HDAC1 (sc-7872), anti-HDAC2 (sc-7899), anti-Ku80 (sc-5280), anti-RbAp46/48 (sc-373873) from Santa Cruz Biotechnology; anti-MBD3 (14540), anti-H2AK119ub (8240), anti-H2BK120ub (5546), anti-γH2AX (9718P), anti-BMI1 (6964) from Cell Signaling Technology; anti-γH2AX (05-636), anti-H2AK15ub (MABE1119), anti-H2A (ABE327), anti-H2B (MABE15), anti-FK2 (04-263) from Merck Millipore; anti-H3ac (39139) from Active Motif; anti-HA (M180-3) from MBL; anti-BRCA1 (22362-1-AP), anti-USP44 (15521-1-AP), anti-Lamin B1 (66095-1-Ig) from Proteintech; anti-53BP1(612522) from BD; anti-PARP1 (A0942), anti-USP3 (A15769), anti-USP12 (A5201), anti-USP22 (A16297) anti-USP30 (A12862) from Abclonal; anti-USP43 (AP14283b) from ABGENT. VP16, CPT, MMC, MMS, PJ-34, 4-OHT, anti-FLAG M2 affinity gel, FLAG peptide were from Sigma, and phosphatase inhibitor was from Applygen, protease inhibitor cocktail was from Roche Applied Science. Protein A/G Sepharose CL-4B beads were from Amersham Biosciences, NuPAGE 4-12% Bis-Tris gel was from Invitrogen, and silver-stained kit was from Pierce.

### RNA interference

The mixture of the siRNA were transfected into cells using Lipofectamine RNAi MAX (Invitrogen) with the final concentration at 10 nM. The sequences used were showed as following: siControl, 5′-UUCUCCGAACGUGUCACGU-3′; siUSP11-1, 5′-GCGCACAGCUGCAUGUCAU-3′; siUSP11-2, 5′-GGACCGUGAUGAUAUCUUC-3′; siUSP11-3, 5′-GAAGAAGCGUUACUAUGAC-3′; siUSP11-3′UTR, 5′-GCCCUGUACCUUCUGCUGUTT-3′; siMi-2β-1, 5′-CCAAGGACCUGAAUGAUGA-3′; siMi-2β-2, 5′-CAAAGGUGCUGCUGAUGUA-3′; siMTA2-1, 5′-CAAAGUCUCUCUCCUUACAUU-3′; siMTA2-2, 5′-UGAACAAGACAGAGCUCAATT-3′; siHDAC2-1, 5′-GCGGAUAGCUUGUGAUGAA-3′; siHDAC2-2, 5′-GCAAAGAAAGCUAGAAUUG-3′; siBRCA1-1, 5′-GGAACCTGTCTCCACAAAG-3′; siBRCA1-2, 5′-GATAGTTCTACCAGTAAA-3′; siKu80-1, 5′-GCGAGUAACCAGCUCAUAAUU-3′; siKu80-2, 5′-AAGAGCUAAUCCUCAAGUCUU-3′; siPARP1-1, 5′-GCATGATTGACCGCTGGTA-3′; siPARP1-2, 5′-GATAGAGCGTGAAGGCGAA-3′; siBMI1-1, 5′-GACAUUGCAUCUGAUCUGU-3′; siBMI1-2, 5′-ACAGAUCAGAUGCAAUGUC-3′; siRNF20-1, 5′-GAUGCAAAUUUCAAGCUCA-3′; siRNF20-2, 5′-GACAGAUCUUCUUCAGGAA-3′. Cells were harvested 72 h later according to the purpose of the experiments.

### Lentiviral production and infection

The generation of the pLKO.1-shUSP11 lentiviruses was conducted according to a protocol described by Addgene (http://www.addgene.org/tools/protocols/plko/#E). They were co-transfected into the packaging cell line HEK293T. The sequences used were showed as following: shControl, 5′-GAATCGTCGTATGCAGTGAAA-3′; shUSP11-1, 5′-CCGTGATGATATCTTCGTCTA-3′; shUSP11-2, 5′-CCGATTCTATTGGCCTAGTAT-3′; shUSP11-3, 5′-CCCTCCCTTCTAGTCTTTATT-3′. Viral supernatants were collected 48 h later, clarified by filtration, and concentrated by ultracentrifugation. The concentrated virus was used to infect 5 × 10^5^ HEK293T, U2OS or HeLa cells (20–30% confluent) in a 60 mm dish with 5 μg/ml polybrene. Infected cells were selected by 2 μg/ml puromycin (Merck Millipore).

### Immunopurification and mass spectrometry

HEK293T cells stably expressing FLAG-USP11 were washed twice with cold PBS, scraped, and collected by centrifugation at 800 g for 5 min. Cellular extracts were prepared using lysis buffer (150 mM NaCl, 0.3% NP-40, 1 mM DTT, 5 mM EDTA, and 50 mM Tris–HCl, pH 7.4) containing protease inhibitor cocktail. Anti-FLAG immunoaffinity resin was prepared according to the manufacturer's protocol. Cell lysates were applied to the immunoaffinity resin to enable adsorption of the protein complex. After binding, the resin was washed with cold PBS plus 0.3% NP-40. FLAG peptide was applied to the resin to elute the FLAG-tagged protein-associated complex. The eluates were collected and resolved on NuPAGE 4–12% Bis–Tris gel, silver-stained and subjected to LC–MS/MS sequencing and data analysis.

### Fast protein liquid chromatography (FPLC)

Cellular extracts were prepared using lysis buffer containing protease inhibitor. Approximately 5 mg protein was concentrated to 0.5 ml using a Millipore Ultrafree centrifugal filter apparatus (3 kD nominal molecular mass limit), and then applied to an 850 × 20 mm Superose 6 size exclusion column (Amersham Biosciences) that had been equilibrated with buffer D containing 1mM dithiothreitol and calibrated with protein standards (Amersham Biosciences, including blue dextran, 2000 kD; thyroglobulin, 669 kD; ferritin, 440 kD; catalase, 158 kD; bovine serum albumin, 43 kD). Elution was carried out at a flow rate of 0.5 ml/min and fractions were collected.

### Histone deubiquitination and histone deacetylation assays

Calf thymus bulk histones (Sigma) were incubated with FLAG-USP11 or FLAG-HDAC2 IPs at 37°C for 90 min in deubiquitination assay buffer (50 mM NaCl, 1mM DTT and 50 mM Tris–HCl, pH 7.5) or histone deacetylation assay buffer (150 mM NaCl, 1mM DTT and 10 mM Tris–HCl, pH 8.0). Each reaction was stopped by mixing with SDS loading buffer followed by western blot analysis with antibodies against H2AK119ub, H2BK120ub, or H3ac.

### Subcellular protein fractionation

The separation and preparation of nuclear soluble and chromatin-bound protein extracts from HeLa, HEK293T or U2OS cells were performed using the Subcellular Protein Fractionation Kit for Cultured Cells (Thermo Scientific) according to the manufacturer's instructions.

### Realtime RT-PCR

Total cellular RNAs were isolated from samples with the Trizol reagent (Invitrogen). First strand cDNA synthesis with the Reverse Transcription System (Roche). Quantitation of all gene transcripts was done by qPCR using Power SYBR Green PCR Master Mix and an ABI PRISM 7500 sequence detection system (Applied Biosystems, Foster City, CA) with the expression of GAPDH as the internal control. The primer pairs used were: *USP11*, 5′-GAGAACGGACGGCGATGG-3′ (forward) and 5′-CACAAGGAACCAGCTTTCGC-3′ (reverse); *MTA2*, 5′-GGAGTGGCCTTCGGAACC-3′ (forward) and 5′-ACGTAATCTTTTCGAATCGAGGC-3′ (reverse); *HDAC2*, 5′-ACTATCGCCCCCACGTTTC-3′ (forward) and 5′-AATATCACCGTCGTAGTAGTAGCAG-3′ (reverse).

### Chromatin immunoprecipitation

About 10 × 10^7^ cells were crosslinked with 1% formaldehyde for 10 min at room temperature and quenched by the addition of glycine to a final concentration of 125 mM for 5 min. The fixed cells were resuspended in lysis buffer (1% SDS, 5 mM EDTA, 50 mM Tris–HCl, pH 8.1) in the presence of protease inhibitors and subjected to 3 × 10 cycles (30 seconds on and 30 seconds off) of sonication (Bioruptor, Diagenode) to generate chromatin fragments of ∼300 bp in length. Lysates were diluted in buffer containing 1% Triton X-100, 2 mM EDTA, 150 mM NaCl, 20 mM Tris–HCl (pH 8.1). For immunoprecipitation, the diluted chromatin was incubated with control or specific antibodies (2 μg) for 12 hours at 4°C with constant rotation. 50 μl of 50% protein A/G magnetic beads was then added and the incubation was continued for an additional 3 h. Beads were then washed with the following buffers: TSE I (0.1% SDS, 1% Triton X-100, 2 mM EDTA, 150 mM NaCl and 20 mM Tris–HCl, pH 8.0), TSE II (0.1% SDS, 1% Triton X-100, 2 mM EDTA, 500 mM NaCl and 20 mM Tris–HCl, pH 8.0), buffer III (0.25 M LiCl, 1% NP-40, 1% sodium deoxycholate, 1 mM EDTA, and 10 mM Tris–HCl, pH 8.0), and Tris-EDTA buffer. Between washes, the beads were collected by magnetic stand at 4°C. Then the pulled down chromatin complex together with input were de-crosslinked at 55°C for 6 h in elution buffer (1% SDS, 0.1 M NaHCO_3_). Eluted DNA was purified with PCR purification kit (Qiagen) and analyzed by qPCR using a Power SYBR Green PCR Master Mix (Roche) and an ABI PRISM 7500 sequence detection system (Applied Biosystems). The sequences of the primers used were showed as following: Chr22 proximal , 5′-CCTTCTTTCCCAGTGGTTCA-3′ (forward) and 5′-GTGGTCTGACCCAGAGTGGT-3′ (reverse); Chr22 distal, 5′-TGGCTGGAACTGCTTTCTTT-3′ (forward) and 5′-GGTGAGTGAATGAGCTGCAA-3′ (reverse); Chr1 proximal, 5′-GATTGGCTATGGGTGTGGAC-3′ (forward) and 5′-CATCCTTGCAAACCAGTCCT-3′ (reverse); Chr1 distal, 5′-CGAGATCCAAGGAAGTCGTG-3′ (forward) and 5′-CCCCGGACACTTTAAAAGGA-3′ (reverse); Chr6 proximal, 5′-TGCCGGTCTCCTAGAAGTTG-3′ (forward) and 5′-GCGCTTGATTTCCCTGAGT-3′ (reverse); Chr6 distal, 5′-ACCTGGGATGGGACATATCA-3′ (forward) and 5′-TACCAAGCCTGTCCCTGAAC-3′ (reverse); Control region lacking AsiSI sites, 5′-CCCATCTCAACCTCCACACT-3′ (forward) and 5′-CTTGTCCAGATTCGCTGTGA-3′ (reverse). *RHOA*, 5′-TGAAGAGTTGGCAGTTCGGG-3′ (forward) and 5′-CGGGAACTCCGGGGCTATAA-3′ (reverse); *IGLL1*, 5′-TAGGTTGTGTGTATGTTACTGCT-3′ (forward) and 5′-TTGAGGTTGGTGTTGGGAGA-3′ (reverse); *BMP2*, 5′-TTACCCCACTCCACTCATCCT-3′ (forward) and 5′-AGACTCCCCTGAGAAGCCTG-3′ (reverse); *Ep300*, 5′-TGTTCTATTGGGAGCGGACG-3′ (forward) and 5′-CGTGTGTCCATACGCCCTTA-3′ (reverse); *HOXA9*, 5′-TTCTCTCGACAGCACGACAC-3′ (forward) and 5′-CGAAGGAGCAGCCAACCTAA-3′ (reverse); *HOXA10*, 5′-ACATTCCTCTCCCTGATCGC-3′ (forward) and 5′-TGAGATACCACCCAGGTCCC-3′ (reverse); *HOXC5*, 5′-CAACCTCTGGGTCCGTTCTC-3′ (forward) and 5′-CGGGCGAGCGAATTAACAGA-3′ (reverse); *AKT3*, 5′-TATTTGGGTAGGCGTGACTGG-3′ (forward) and 5′-GTCTTCAACTGGCCTGACCT-3′ (reverse).

### Laser microirradiation and X-ray irradiation

For time-lapse imaging of living cells, cells grown on a dish with thin glass bottom (NEST) were transfected with GFP-tagged expression constructs. 24 hours after transfection, cells were locally irradiated with a 365 nm pulsed nitrogen ultraviolet laser (16 Hz pulse, 45% laser output) generated from the micropoint system (Andor Technology). To measure the protein accumulations at laser-generated DSBs, the mean fluoresecence intensity within the regions of interest (ROI) was estimated by ImageJ. The intensity values were background subtracted and the ratio of intensity within the microirradiated nuclear area to non-microirradiated area was calculated.

For laser-induced DNA DSBs and immunofluorescent assays, cells were grown on LabTek II chamber slides (Thermo Fisher Scientific) for 24 h before induction of DNA damage (ultraviolet-A laser, λ = 355 nm, 40% energy) by a Zeiss Observer.Z1 inverted microscope with a PALM MicroBeam laser microdissection workstation. After irradiation, cells were incubated at 37°C for appropriate time and processed for immunofluorescent staining.

For X-ray irradiation, IR was delivered by an X-ray generator at different dosage (RS2000 PRO, 160 kV, 25 mA; Radsource Corporation). After irradiation, the cells were incubated at 37°C for an appropriate time and processed for western blotting, immunostaining, MNase sensitivity assay or clonogenic survival assay.

### Immunofluorescent staining

Cells were washed with PBS for three times, fixed in 4% paraformaldehyde for 10 min, and permeabilized with PBS containing 0.2% Triton X-100 for 10 min at room temperature. Cells were incubated with primary antibodies over night at 4°C and secondary antibodies coupled to Alexa Fluor 488 (ZhongShanJinQiao, ZF-0511 or ZF-0512) or Alexa Fluor 594 (ZhongShanJinQiao, ZF-0516 or ZF-0513). The cells were then washed for four times, and a final concentration of 0.1 μg/ml DAPI (Sigma) was included to stain nuclei. Images were acquired with a LSM880 laser scanning confocal system (Zeiss). To avoid bleed-through effects in double-staining experiments, each dye was scanned independently in a multi-tracking mode.

### Proximity ligation assay (PLA)

Proximity ligation assay was performed using the Duolink PLA Fluorescence Kit (Sigma) according to the manufacturer's instructions. U2OS cells were subjected to a ultraviolet-A laser (λ = 355 nm, 40% energy) and collected at different time points after microirradiation. Cells were fixed in 4% paraformaldehyde for 10 min, and permeabilized with PBS containing 0.2% Triton X-100 for 10 min at room temperature. Cells were incubated with Duolink blocking solution at 37°C for 1 h followed by primary antibodies (anti-USP11 and anti-MTA2; anti-USP11 and anti-HDAC2) over night at 4°C. Then, cells were incubated with proximity ligation assay probes PLUS and MINUS for 1 h at 37°C and ligation–ligase solution for 30 min at 37°C. After ligation, cells were incubated with an amplification polymerase solution for 100 min at 37°C. Cells were counterstained for 1 h at room temperature with secondary antibodies coupled to Alexa Fluor 594 to stain MTA2 or HDAC2, and a final concentration of 0.1 μg/ml DAPI was included to stain nuclei. Images were acquired with a LSM880 laser scanning confocal system.

### HR and NHEJ reporter assay

To examine distinct outcomes of chromosomal DSB repair, two cell lines containing *GFP*-based reporters with recognition sites for the rare-cutting endonuclease I-SceI were utilized. DR-GFP-U2OS cells, in which *SceGFP* cassette is interrupted by a single I-SceI site and cleavage of the I-SceI sites leads to the restoration of GFP gene through HR, were used for measurement of HR repair efficiency (45). EJ5-HEK293 cells, in which contain a promoter that is separated from the rest of a GFP expression cassette by a marker gene (*puro*) that is flanked by two I-SceI sites, were used for measurement of NHEJ repair efficiency. The excision of the two I-SceI sites followed by NHEJ eliminates the translation start codon of the otherwise non-sense transcript and enables the reading frame shift and subsequently expression of the GFP gene (46,47). Accordingly, the GFP-marked repair outcome is measured by transiently expressing I-SceI, culturing the cells to allow completion of repair, then determining the percentage of GFP positive cells by FACS analysis. In particular, DR-GFP-U2OS and EJ5-HEK293 were treated with siRNAs against USP11, BRCA1, Ku80 and/or RNF20, BMI1 48 h prior to HA-I-SceI transfection. 24 hours after transfection, cells were trypsinized, washed with PBS and collected at least 10^4^ cells with FACSCalibur. The knockdown efficiency of USP11, RNF20, BMI1, BRCA1 or Ku80 siRNAs and expression levels of I-SceI, USP11, or USP11/C318S plasmids were validated by western blotting.

### Flow cytometry analysis

The cells were harvested in PBS, and fixed in suspension with 70% ethanol for 2 h while rotating at 4°C. The fixed cells were washed with PBS, incubated with RNase A in PBS for 30 min at 37°C and then stained with 50 mg/ml propidium iodide (PI). The percentage of GFP-positive cells or cell cycle data were collected using FACSCalibur and analyzed with FlowJo.

### MNase sensitivity assay

U2OS cells transfected with siRNAs or/and expression plasmids were exposed to X-ray IR (10 Gy) and then incubated at 37°C to recover for appropriate time. 3–5 million cells were washed with cold PBS, resuspended in ice-cold NP-40 cell lysis buffer (10 mM NaCl, 3 mM MgCl_2_, 0.4% NP-40, 0.15 mM Spermine, 0.5 mM Spermidine and 10 mM Tris–HCl, pH 7.4) in the presence of protease inhibitors and incubated on ice for 5 min. The lysate was cleared with centrifugation at 2000 g for 5 min at 4°C. The pellet was then resuspended in 50 ml glycerol buffer (5 mM MgAc_2_, 25% (v/v) glycerol, and 10 mM Tris–HCl, pH 7.4), mixed with equal volume of 2× MNase buffer (50 mM KCl, 8 mM MgCl_2_, 2 mM CaCl_2_, and 100 mM Tris–HCl, pH 7.4), and incubated at 37°C for 10 min with 2.5 U/ml micrococcal nuclease (NEB). The reaction was stopped by adding EDTA at the final concentration of 5 mM. Genomic DNA was purified and separated by electrophoresis in 1.2% agarose gel.

### Analysis of chromosomal aberrations

U2OS clones with control or USP11 stably depleted were generated by lentivirus-delivered shRNA were treated with DMSO or 2 μM CPT for 12 h, exposed to 1 μg/ml colcemid (Selleck) for 4 h and then swollen using 75 mM KCl for 30 min at 37°C. After fixing in methanol/acetic acid (3:1) (v/v) for 20 min twice, cells were dropped onto ice-cold wet slides, air dried, and stained with 5% Giemsa for 5 min. The number of chromosome aberrations were scored in 50 metaphases per sample.

### Apoptosis assay

For induction of apoptosis, 1 × 10^6^ cells were seeded into 60-mm petri dishes, and allowed to attach for 24 h after which cells were treated with DMSO, 1 μM CPT or 40 nM VP16 for 48 h. The cells were trypsinized, washed with PBS, resuspended in Annexin V binding buffer and then stained with FITC Annexin V and propidium iodide for 10 min in the dark at room temperature according to the instructions of Annexin V-FITC Apoptosis Detection Kit (BD Pharmingen). A minimum of 10^4^ cells per sample were acquired and analyzed using the FACSCalibur flow cytometer.

### Clonogenic survival assay

HeLa or U2OS cells with control or stably USP11-depleted were plated in 6-well plates in triplicates (500 cells per well) for 24 h, and were subsequently treated with X-ray IR, CPT or VP16 for 24 h before growing in colonies for 5 days. The cells were then washed with PBS, fixed with 4% formaldehyde for 10 min and stained with crystal violet (0.1% w/v) for 20 min. The number of colonies per well was counted, and surviving fraction for given treatments were calculated on the basis of the survival rates of untreated cells.

### Statistical analysis

The data were analyzed by a two-tailed unpaired Student's *t*-test for two-group comparisons, or ANOVA with Bonferroni's correction for multiple comparisons (GraphPad Prism software, version 5.01) and expressed as mean ± SD unless otherwise indicated. *P* < 0.05 was considered to be statistically significant.

## RESULTS

### USP11 acts as a histone deubiquitinase to catalyze H2AK119 and H2BK120 deubiquitination

**Figure 1.**
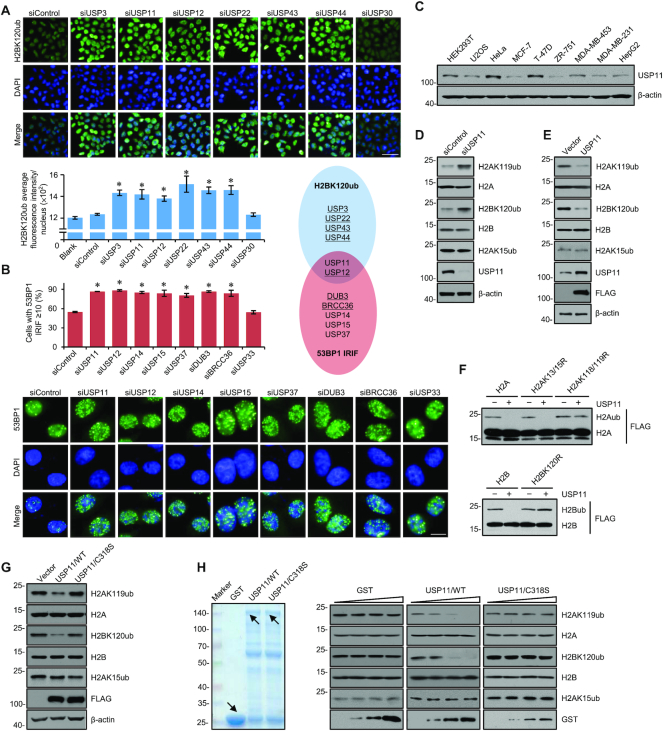
USP11 Acts as a Histone Deubiquitinase to Catalyze H2AK119 and H2BK120 Deubiquitination. (**A**) HeLa cells were transfected with siRNA pools against each of the 81 corresponding DUBs and followed by immunofluorescent staining with antibodies against H2BK120ub (green). DAPI staining was included to visualize the nucleus (blue). The representative images in each group are shown. Bar, 50 μm. High-content automated image-processing system was applied to determine the mean immunofluorescent intensity per cell. More than 500 cells were analyzed in each group. Error bars represent mean ± SD for triplicate experiments (*P < 0.05). (**B**) siDUBs-treated HeLa cells were exposed to X-ray-generated IR (10 Gy), collected at 8 h after irradiation and immunofluorescently stained with antibodies against 53BP1 (green). DAPI staining was included to visualize the nucleus (blue). The representative images in each group are shown. Bar, 10 μm. The number of 53BP1 IRIF per cell was counted by high content image-processing system. More than 500 cells were analyzed in each group. Error bars represent mean ± SD for triplicate experiments (*P < 0.05). Underlined DUBs in Venn diagram represent these that are previously reported to be linked to H2BK120 deubiquitination (blue) or 53BP1 IRIF (red). (**C**) Western blotting analysis of USP11 expression in multiple cell lines with antibodies against USP11. (**D**) Western blotting analysis of the level of the indicated histone marks or proteins in HeLa cells treated with control or USP11 siRNAs. (**E**) Western blotting analysis of the level of the indicated histone marks or proteins in HEK293T cells transfected with vector or FLAG-USP11 expression construct. (**F**) FLAG-tagged histone plasmids were transfected into HEK293T cells. Mononucleosomes were then purified using anti-FLAG antibody and incubated with bacterially expressed GST-USP11 followed by western blotting analysis with anti-FLAG antibody. (**G**) Western blotting analysis of the level of the indicated histone marks or proteins in HEK293T cells transfected with vector, FLAG-USP11/WT or FLAG-USP11/C318S expression construct. (**H**) *In vitro* deubiquitination assays with bacterially expressed GST-fused USP11 or USP11/C318S protein and calf thymus histones. Coomassie brilliant blue staining of the GST or GST-fused proteins was shown with *arrows* (left). The reaction was analyzed by western blotting with antibodies against the indicated histone marks or proteins (right).

It is becoming increasingly clear that histone modifications such as ubiquitination and subsequent recruitment of DNA repair proteins are the integral parts of the regulatory network in response to DNA damages (5,17,18,29), and a number of DUBs have been implicated in DNA repair process (14,26,27,48–52). In order to have a general view of the multitude of DUBs that potentially participate in mammalian DSB response, we systematically screened 81 DUBs for their role in H2BK120 deubiquitination or in ionizing radiation-induced foci (IRIF) formation of 53BP1, a well-known DDR factor that is recruited to DNA damage sites and forms readily visualized IRIF (53). We employed 81 siRNA pools to individually knock down each of the corresponding DUB in HeLa cells. Examination of the immunofluorescent intensity of H2BK120ub and 53BP1 IRIF formation in undamaged cells by high-content imaging system showed positive results for several DUBs, including USP3, USP12, USP22, USP43 and USP44, which has been reported to be able to deubiquitinate H2BK120 (54–58), validating our screening methodology. Interestingly, the average immunofluorescent intensity of H2BK120ub markedly increased upon the depletion of USP11, whereas depletion of USP30 had a limited effect on the immunofluorescent intensity of H2BK120ub (Figure 1A). The similar results were obtained by western blotting analysis (Supplementary Figure S1A). Meanwhile, analysis of IRIF formation of 53BP1 in siDUBs-treated HeLa cells showed that, in addition to USP17 and BRCC36, which are known to impede the accumulation of 53BP1 (13,59), depletion of USP11, USP12, USP14, USP15 or USP37 all led to an increase in 53BP1 IRIF, whereas knockdown of USP33 had a marginal effect on 53BP1 IRIF (Figure 1B and Supplementary Table S1). These observations indicate that USP11 and USP12 are potentially associated with DDR by targeting H2BK120ub. Given that USP12 has been reported to deubiquitinate H2A and H2B in *Xenopus* development (55), we thus focused on our subsequent study on USP11.

To verify the role of USP11 in histone deubiquitination, we first tested USP11 protein expression profile in multiple cell lines. The results showed that USP11 is highly expressed in HeLa cells, while its expression in HEK293T cells is relatively low (Figure 1C). HeLa cells were thus treated with USP11 siRNAs and HEK293T cells were transfected with USP11 expression plasmids. Western blotting analysis of acidly extracted histones with antibodies against ubiquitinated H2AK15, H2AK119, or H2BK120, the prevalent ubiquitination sites in histone (20,60–62), showed that USP11 knockdown resulted in an increase in the levels of both H2AK119ub and H2BK120ub, while USP11 overexpression led to a decrease in the ubiquitination levels of both H2BK120 and H2AK119 (Figure 1D and E). No evident changes were observed for the level of H2AK15ub (Figure 1D and E). Given that BMI1 is a well-studied ubiquitin ligase for H2AK119 and RNF20 is an ubiquitin ligase for H2BK120 (60,63), we found BMI1 knockdown or RNF20 depletion led to a decrease in H2AK119ub or H2BK120ub respectively (Supplementary Figure S1B), validating the specificity of antibodies. Considering direct contribution of H2AK15ub to 53BP1 recruitment to DSBs (19) and involvement of H2AK119ub in DSB repair (64), we then utilized histone substitution mutants to confirm the targets of USP11. To this end, FLAG-tagged H2A, H2B, H2AK13/15R, H2AK118/119R, H2BK120R plasmids were transfected into HEK293T cells followed by mononucleosome purification with anti-FLAG. Ubiquitylated species were then incubated with bacterially expressed GST-USP11 followed by western blotting analysis. The results showed USP11 could efficiently remove ubiquitin from H2A, H2B, H2AK13/15R nucleosomes, but had no evident effect on ubiquitination of H2AK118/119R or H2BK120R nucleosomes (Figure 1F), indicating that H2AK13/15ub are not the catalytic sites for USP11. Together, these observations indicate that USP11 is functionally linked to removal of H2AK119 and H2BK120 ubiquitination.

To support this, we created a USP11 mutant, USP11/C318S, by site-directed mutagenesis, which is predicted, based on the structural comparison of the putative USP domains of DUBs (42), to interrupt the catalytic core. Western blotting analysis of histones extracted from HEK293T cells revealed that overexpression of wild-type USP11 resulted in significant decreases in H2BK120ub and H2AK119ub levels, whereas overexpression of USP11/C318S had no evident effect on the levels of H2BK120ub and H2AK119ub (Figure 1G). Moreover, *in vitro* deubiquitination assays with bacterially expressed GST-USP11 or GST-USP11/C318S and calf thymus histones followed by western blotting showed that while recombinant wild-type USP11 could efficiently remove ubiquitin from H2BK120 and H2AK119 in a dose-dependent manner, USP11/C318S lost the deubiquitination activity toward H2BK120 and H2AK119 (Figure 1H). Together, these results support a notion that USP11 acts to remove H2BK120 and H2AK119 deubiquitination through its deubiquitinase activity.

### USP11 is physically associated with the NuRD complex

**Figure 2.**
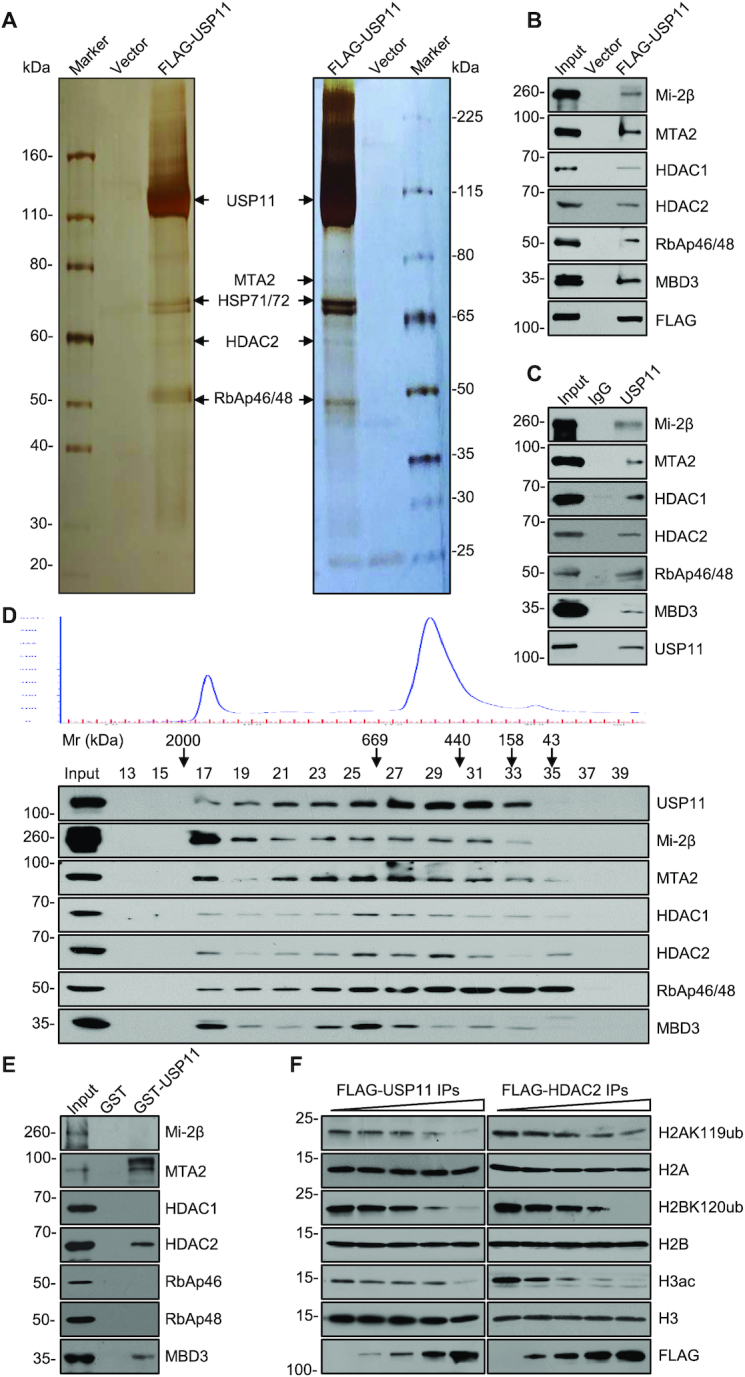
USP11 Is Physically Associated with the NuRD Complex. (**A**) Cellular extracts from HEK293T stably expressing vector or FLAG-USP11 were prepared and subjected to affinity purification with anti-FLAG M2 affinity column and eluted with FLAG peptides. The eluates were resolved by SDS-PAGE and silver-stained. The protein bands were retrieved and analyzed by mass spectrometry. The experiment was repeated three times, and the results from two experiments are shown. (**B**) Whole-cell lysates from HEK293T cells transfected with FLAG-USP11 were prepared and co-immunoprecipitation experiments were performed with anti-FLAG followed by immunoblotting with antibodies as indicated. (**C**) Whole-cell lysates from HEK293T cells were immunoprecipitated with antibodies against USP11 or IgG followed by immunoblotting with the antibodies as indicated. (**D**) Whole-cell lysates from HEK293T cells were extracted, concentrated, and fractionated on Superose 6 size exclusion columns. Chromatographic eluate profiles and the eluate positions of calibration proteins with known molecular masses (kDa) were indicated. An equal volume of eluates from each chromatographic fraction was analyzed by western blotting. (**E**) GST pull-down assays were performed with GST or GST-fused USP11 and *in vitro* transcribed/translated proteins as indicated. (**F**) Cellular extracts from HEK293T cells overexpressing FLAG-USP11 (left) or FLAG-HDAC2 (right) were immunopurified with anti-FLAG M2 affinity column and eluted with FLAG peptides. Calf thymus histones were incubated with the eluates with different doses in histone deubiquitination or histone deacetylation assay buffer. The reaction was analyzed by western blotting with antibodies as indicated.

To explore the cellular function of USP11, we next employed affinity purification and mass spectrometry to interrogate the USP11 interactome *in vivo*. To this end, HEK293T cells were stably transfected with FLAG-tagged USP11. Cellular extracts were prepared and subjected to affinity purification with anti-FLAG affinity columns. Mass spectrometric analysis of the USP11-containing protein complex showed that USP11 was co-purified with MTA2, HDAC2, RbAp46/48, all components of the nucleosome remodeling and deacetylase (NuRD) complex (Figure 2A and Supplementary Table S2).

To verify the observation, protein extracts from HEK293T cells overexpressing FLAG-USP11 were co-immunoprecipitation (IP) with anti-FLAG followed by immunoblotting (IB) with antibodies against Mi-2β, MTA2, HDAC1, HDAC2, RbAp46/RbAp48 or MBD3. The results confirmed the interaction of USP11 with the tested components of the NuRD complex (Figure 2B). In addition, total proteins from HEK293T cells were extracted and co-IP experiments were performed with anti-USP11 followed by IB with antibodies against endogenous Mi-2β, MTA2, HDAC1, HDAC2, RbAp46/RbAp48 or MBD3. These experiments also confirmed the interaction of USP11 with the NuRD components (Figure 2C).

To further support the physical interaction of USP11 with the NuRD complex *in vivo*, proteins from HEK293T cells were fractionated by fast protein liquid chromatography (FPLC) with Superose 6 columns. Native USP11 from HEK293T cell extracts was eluted with an apparent molecular mass much greater than that of the monomeric protein; USP11 immunoreactivity was detected in chromatographic fractions with a relative symmetric peak centered between ∼158 and ∼2000 kDa. Moreover, the elution pattern of USP11 largely overlapped with that of the NuRD complex proteins including Mi-2β, MTA2, HDAC1/2, RbAp46/48 and MBD3 (Figure 2D). Together, these results support the existence of the USP11/NuRD complex *in vivo*.

To further consolidate the interaction between USP11 and the NuRD complex and to investigate the molecular details for this interaction, GST pull-down assays were performed with bacterially expressed GST or GST-fused USP11 and *in vitro* transcribed/translated individual components of the NuRD complex. These experiments revealed that USP11 was capable of interacting with MTA2, HDAC2 and MBD3 directly, but not with the other components of the NuRD complex that we tested (Figure 2E), suggesting that the association of USP11 with the NuRD complex is through direct binding of USP11 to MTA2, HDAC2 and MBD3. Collectively, these experiments confirmed that USP11 is physically associated with NuRD complex *in vivo* and *in vitro*.

To explore the functional significance of the physical interaction between USP11 and the NuRD complex, USP11-containing protein complex as well as the NuRD complex were immunoprecipitated with anti-FLAG from HEK293T cells overexpressing FLAG-USP11 or FLAG-HDAC2, respectively. The IPs were incubated with calf thymus histones, and the enzymatic activities of the immunocomplexes were analyzed by western blotting. As expected, the USP11-containing complex possessed an enzymatic activity that led to significant decreases in the levels of H2BK120ub and H2AK119ub. Remarkably, the USP11-containing complex also exhibited a dose-dependent deacetylase activity toward H3 (Figure 2F). Analogously, the HDAC2-containing NuRD complex possessed not only deacetylase activity for H3 but also deubiquitinase activity for H2BK120 and H2AK119 (Figure 2F), further supporting the molecular interaction between USP11 and the NuRD complex. Moreover, USP11 knockdown had no obvious effect on steady-state levels of MTA2 and HDAC2 proteins (Supplementary Figure S2A), and HDAC2 depletion did not led to evident changes of *USP11* (Supplementary Figure S2B), excluding the possibility that USP11 regulates the protein stability of NuRD components and HDAC2 affects the transcription of *USP11*.

### The USP11/NuRD complex is recruited to DNA break sites upon DNA damage

**Figure 3.**
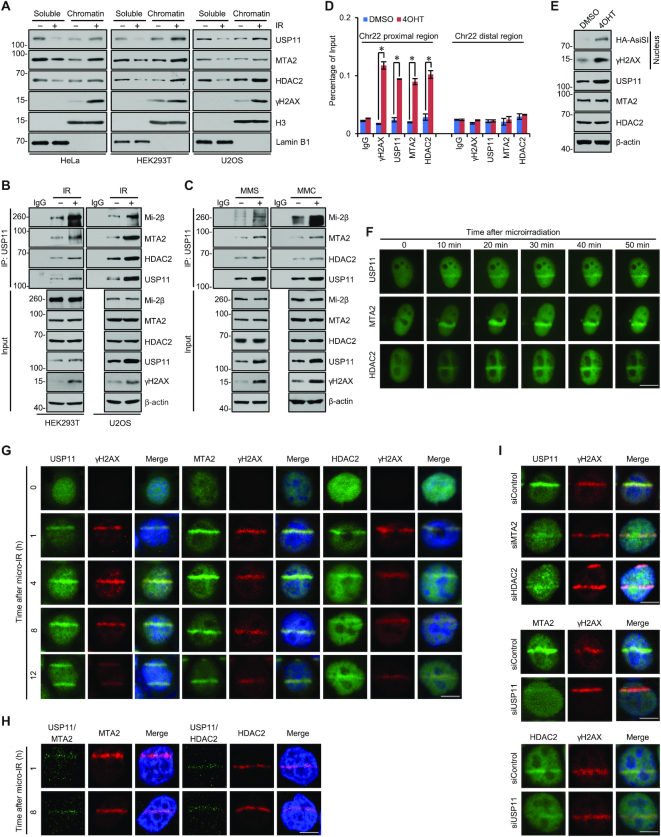
The USP11/NuRD Complex Is Recruited to DNA Break Sites upon DNA Damage. (**A**) HeLa, HEK293T, or U2OS cells exposed to X-ray IR (6 Gy) were collected at 1 h post IR. Nuclear proteins were extracted and split into chromatin-free and chromatin-bound fractions for western blotting analysis with the indicated antibodies. (**B**) HEK293T or U2OS cells were collected at 8 h post IR (6 Gy), and cellular extracts were immunoprecipitated and then immunoblotted with antibodies as indicated. (**C**) HEK293T cells were cultured in methyl methanesulfonate (MMS, 0.001%, 4 h) or mitomycin C (MMC, 1 μg/ml, 4 h). Cellular extracts were immunoprecipitated and then immunoblotted with antibodies as indicated. (**D**) U2OS cells stably expressing HA-AsiSI were treated with DMSO or 1 μM 4OHT for 4 h. qChIP experiments were performed using antibodies as indicated with primers that cover the DNA sequences flanking the AsiSI site and the break distal regions in Chr22. Each bar represents the mean ± SD for triplicate experiments (*P < 0.05). (**E**) 4OHT-induced AsiSI nuclear localization and γH2AX induction were monitored by western blotting with the indicated antibodies. (**F**) U2OS cells transfected with the GFP-tagged USP11, MTA2 or HDAC2 plasmids were subjected to a 365 nm pulsed nitrogen ultraviolet laser (16 Hz pulse, 45% laser output) using the micropoint system and analyzed by fluorescent microscopy for the accumulation in DSBs. Bar, 10 μm. (**G**) U2OS cells were subjected to a ultraviolet-A laser (λ = 355 nm, 40% energy) and immunofluorescent analysis with the indicated antibodies at different time points after microirradiation. γH2AX was used as a positive control. Bar, 5 μm. (**H**) U2OS cells were subjected to a ultraviolet-A laser (λ = 355 nm, 40% energy) and collected at different time points after microirradiation. Duolink proximity ligation assay (PLA) was performed with anti-USP11and anti-MTA2 or anti-USP11 and anti-HDAC2 antibodies. PLA signals were shown in green, staining of MTA2 or HDAC2 was shown in red, and nuclei were stained blue by DAPI. Bar, 5 μm. (**I**) U2OS cells pre-treated with MTA2, HDAC2, or USP11 siRNAs were subjected to ultraviolet-A laser (λ = 355 nm, 40% energy), and immunofluorescent analyses were performed at 4 h after micro-IR. γH2AX was used as a positive control. Bar, 5 μm.

Given that ubiquitination of histone H2A and H2B is important for timely initiation of DNA repair and the NuRD complex is implicated in DSB repair and checkpoint activation in response to IR (23,60,65–71). We next investigated whether and how USP11 might co-opt the NuRD complex to participate in DNA damage response. To this end, we first examined the nuclear redistribution of USP11 in relationship to that of the NuRD complex after DNA damage. Nuclear proteins were extracted from HeLa cells pre-treated with X-ray IR and split into chromatin-free and chromatin-bound fractions. We detected IR-triggered phosphorylation of H2AX, an immediate target of ATM (72), as expected. Upon exposure of IR, the protein levels of USP11, MTA2 and HDAC2 in the soluble fraction decreased while their accumulations in chromatin-bound fraction increased (Figure 3A). Similar results were obtained in HEK293T and U2OS cells (Figure 3A), supporting a notion that the USP11/NuRD complex is involved in DDR. Indeed, the protein expression of USP11 increased and the recruitment of the USP11/NuRD complex was elevated in HEK293T and U2OS cells upon IR exposure (Figure 3B). Similarly, treatment of HEK293T cells with alkylating DNA-damaging agents methyl methanesulfonate (MMS) or DNA interstrand crosslinking agent mitomycin C (MMC) resulted in an increase in the steady-state level of USP11 protein and the formation of the USP11/NuRD complex (Figure 3C), implying an important role for the USP11/NuRD complex in DNA damage response.

To further support the recruitment of the USP11/NuRD complex to the vicinity of DSBs, we utilized a DIvA (DSB inducible via AsiSI) system, in which treatment with 4-hydroxytamoxifen (4OHT) triggers nuclear translocation of AsiSI endonuclease, allowing that DSBs are induced at AsiSI-targeted sequence across the human genome (73). Quantitative ChIP (qChIP) analysis showed that, similar to γH2AX, USP11, MTA2, and HDAC2 were enriched around the proximal break sites, but not the distal region about 2 Mb away from the break site upon 4OHT-induced AsiSI activation in U2OS cells stably expressing HA-AsiSI plasmids (Figure 3D). The working efficiency of the AsiSI system was validated by the nuclear localization of AsiSI and induction of γH2AX in response to 4OHT, as showed by western blotting (Figure 3E). Similar results were obtained from other two AsiSI-induced DSB sites and their distal regions (Supplementary Figure S3A, B), indicating that the recruitment of USP11 and NuRD in DSBs is a general feature of DNA damage repair.

Next, time-lapse imaging analysis of U2OS cells stably expressing GFP-USP11, GFP-MTA2, or GFP-HDAC2 was performed to examine the recruitment kinetics of the USP11/NuRD complex in DSBs. The enrichment of USP11, MTA2, and HDAC2 to laser-induced DNA lesions was observed at 10 min and peaked at 30 min after ultraviolet laser micro-irradiation (Figure 3F). In addition, lower doses of laser micro-irradiation and longer recovery time measurement in U2OS cells showed that USP11, MTA2, and HDAC2 co-localized with γH2AX and co-enriched in sites of laser-induced DNA breaks at 1 h after micro-irradiation and persisted to 12 h (Figure 3G). In addition, Duolink proximity ligation assay (PLA) by immunofluorescent microscopy showed that USP11, MTA2, and HDAC2 co-localized at laser-inflicted DNA damage tracks (Figure 3H). Moreover, depletion of MTA2 or HDAC2 led to a decreased recruitment of USP11 to laser-inflicted DNA damage tracks, and USP11 knockdown hindered NuRD enrichment in DSB sites (Figure 3I), suggesting that USP11 and the NuRD components had a synergistic promoting effect on USP11/NuRD relocalization to DNA damage sites.

The NuRD complex is known to repress gene transcription after DNA damage in a poly(ADP-ribose)-dependent manner (70,74). To further support the argument that USP11 is functionally associated with NuRD complex and to investigate the molecular insight into the recruitment of USP11 to DNA damage sites, whole-cell lysates from U2OS cells exposed to IR were prepared and co-immunoprecipitation experiments showed that IR exposure resulted in an enhanced interaction between PARP1 and USP11, and production of highly PARylated USP11-associated immunocomplex (Supplementary Figure S4A). In addition, PARP1 knockdown led to a diminished chromatin binding of USP11 (Supplementary Figure S4B). Meanwhile, U2OS cells stably expressing HA-AsiSI were treated with PARP1 siRNAs. DIvA followed by qChIP analysis showed that PARP1 knockdown resulted in a decreased binding of not only MTA2 and HDAC2 but also USP11 in DSBs upon 4OHT treatment (Supplementary Figure S4C). Consistently, immunofluorescent imaging analysis showed PARP1 depletion led to an decreased enrichment of USP11, MTA2, or HDAC2 in laser-induced DNA lesions (Supplementary Figure S4D), and Duolink PLA showed that PARP1 depletion hindered co-localization of USP11 and NuRD components at laser-inflicted DNA damage tracks (Supplementary Figure S4E). These results demonstrated that the recruitment of USP11 to damaged chromatin is dependent on PARP1, further supporting the physical association and functional connection between USP11 and the NuRD complex in response to DNA damage.

### Crosstalk between histone deubiquitination and deacetylation in DNA damage response

**Figure 4.**
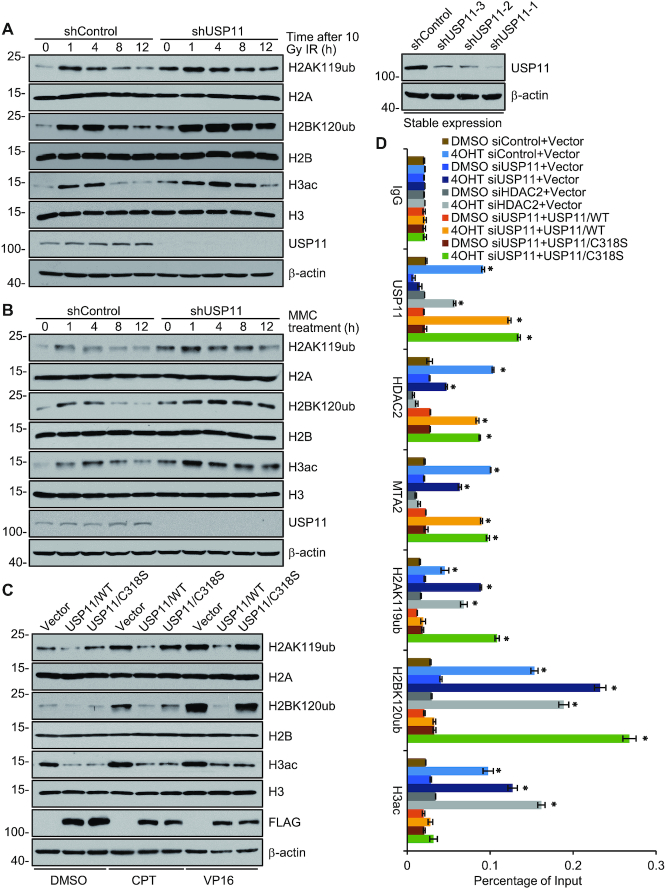
Crosstalk between Histone Deubiquitination and Deacetylation in DNA Damage Response. (**A**) U2OS clones with control or USP11 stably depleted were generated by lentivirus-delivered shRNA and exposed to 10 Gy X-ray IR. Histones were acidly extracted at different time points after IR for western blotting analysis with the indicated histone marks or proteins (left). The efficiency of USP11 knockdown was monitored by western blotting with antibodies against USP11 and shUSP11-1 clone was used (right). (**B**) U2OS clones with control or USP11 stably depleted were treated with 4 μg/ml MMC for the indicated time periods, and histone marks or proteins were detected by western blotting. (**C**) Vector, FLAG-USP11 or FLAG-USP11/C318S overexpressing U2OS cells were treated with 1 μM CPT or 40 nM VP16 for 8 h followed by histone extraction and western blotting analysis with the indicated antibodies. (**D**) U2OS cells stably expressing HA-AsiSI were co-transfected with siRNAs targeting USP11 3′-UTR, or HDAC2, and/or wild-type USP11 or USP11/C318S expression plasmids in the absence or presence of 1 μM 4OHT. qChIP experiments were performed using antibodies and primers that cover the DNA sequences flanking the AsiSI site in Chr22. Each bar represents the mean ± SD for triplicate experiments (*P < 0.05).

To further understand the involvement of the USP11/NuRD complex in DNA damage response, U2OS cells were treated with X-ray IR for histone extraction at different time points after IR. Western blotting analysis showed that the levels of H2AK119ub and H2BK120ub peaked at 1–4 h after IR exposure and gradually decreased thereafter till 12 h (Figure 4A), a manner consistent with previous reports (14,27,52). However, when USP11 was depleted, the levels of H2AK119ub and H2BK120ub increased at all-time points after IR treatment, and these marks were cleared at a much slower rate compared to control cells (Figure 4A). Interestingly, USP11 knockdown also resulted in an increase in H3ac level and a delay of H3ac clearance, while the levels of H2A, H2B and H3 remained unchanged (Figure 4A). Similar results were obtained with MMC treatment (Figure 4B). These observations imply the crosstalk between histone deubiquitination and deacetylation in DNA damage response, consistent with the physical interaction of USP11 with the NuRD complex.

Next, U2OS cells were transfected with wild-type USP11 or deubiquitinase activity-defective USP11/C318S mutant and treated with DNA damaging reagents camptothecin (CPT) and etoposide (VP16) (75,76). Western blotting showed that overexpression of USP11, but not USP11/C318S, was associated with reduced levels of H2AK119ub and H2BK120ub in responding to CPT and VP16 treatment (Figure 4C). The observation that USP11/C318S overexpression also led to a decrease in the H3ac level support a notion that the effect of USP11 on histone deubiquitination is dependent on the intact enzymatic activity of USP11 and the effect of USP11 on histone deacetylation is through its association with NuRD complex.

To further understand the chromatin engagement of the USP11/NuRD complex during DNA damage response, U2OS cells stably expressing HA-AsiSI were co-transfected with siRNAs targeting USP11 3′-UTR, or HDAC2, and/or USP11 or USP11/C318S expression plasmids in the absence or presence of 4OHT induction. Endonuclease AsiSI assays followed by qChIP analysis showed that USP11 knockdown hindered the recruitments of MTA2 and HDAC2 in DSBs, thereby preventing the clearance of not only H2AK119ub and H2BK120ub, but also H3ac around DNA break regions upon 4OHT treatment (Figure 4D). Analogously, HDAC2 depletion was associated with an increase of not only H3ac, but also H2AK119ub and H2BK120ub in DSBs upon 4OHT treatment (Figure 4D). Meanwhile, overexpression of wild-type USP11 was able to counteract the increase in the levels of H2AK119ub, H2BK120ub and H3ac associated with depletion of endogenous USP11, whereas overexpression of USP11/C318S had no such an effect, although USP11/C318S was effectively recruited to the break regions even with a stronger binding than that of wild-type USP11 (Figure 4D). The expression level of proteins was monitored by western blotting analysis (Supplementary Figure S5A). γH2AX enrichment in the proximal break sites and the known sites enriched for MTA2, H3ac, H2BK120ub or H2AK119ub (73,77–83) have been detected as positive control (Supplementary Figure S5B), and the distal region about 2 Mb away from the AsiSI cleavage and control genomic sequence lacking AsiSI sites have been detected as negative controls (Supplementary Figure S5C). Similar results were obtained with another two known AsiSI-induced DSB sites (Supplementary Figure S5D, E). Moreover, the immunostaining assays with H2BK120ub and FK2 antibodies in micro-irradiated cells treated with USP11 siRNAs were performed, and the results showed USP11 knockdown led to an increase in immunofluorescent intensity of H2BK120ub and conjugated ubiquitin in laser-inflicted DNA damage tracks, while in undamaged region of micro-irradiated cells, USP11 knockdown also resulted in an overall increase in level of H2BK120ub and conjugated ubiquitin (Supplementary Figure S5F), suggesting that histone deubiquitination by USP11 is not merely confined to DNA damage sites. Collectively, these observations indicate that USP11 is required for timely clearance of histone ubiquitination and acetylation in DSB sites in response to DNA damage, and that USP11 does so, dependent on its deubiquitinase activity and through its physical and functional connection with the NuRD complex, although USP11 deubiquitinase activity is dispensable for NuRD recruitment.

### The functional significance of USP11-catalyed histone deubiquitination in DNA damage response

**Figure 5.**
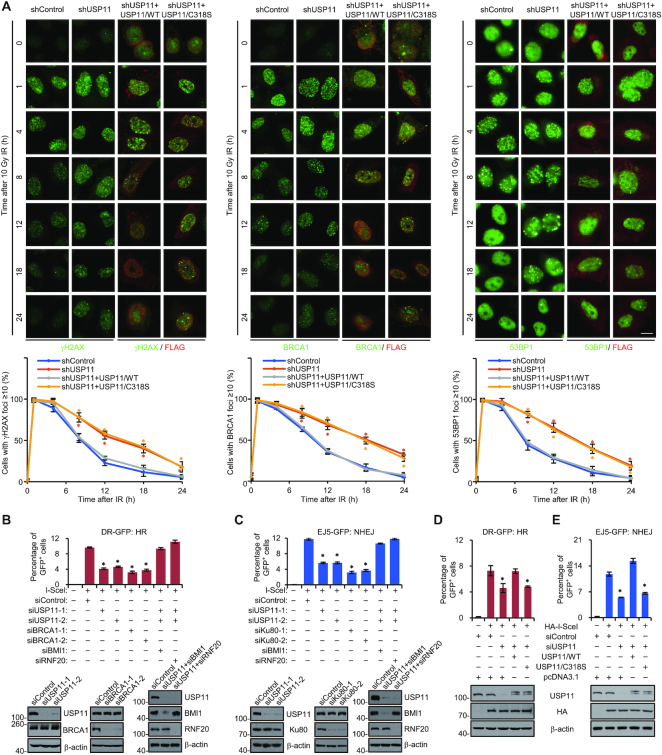
The Functional Significance of USP11-catalyed Histone Deubiquitination in DNA Damage Response. (**A**) U2OS cell clones with control or USP11 stably depleted were generated by lentivirus-delivered shRNA and transfected with FLAG-tagged USP11 or USP11/C318S expression plasmids. These cells were exposed to X-ray IR (10 Gy) and subjected to immunofluorescent staining for γH2AX, BRCA1 or 53BP1 at different time points post IR. High-content immunofluorescent microscope with automatic image processing was applied to determine the number of IRIF per cell. More than 500 cells were analyzed in each group. Bar, 10 μm. The percentage of cells with γH2AX, BRCA1 or 53BP1 IRIF ≥10 per cell was quantified (*P < 0.05). Each bar represents the mean ± SD for triplicate experiments. (**B**) DR-GFP-U2OS cells treated with USP11, and/or RNF20, BMI1, BRCA1 siRNAs were transfected with I-SceI, and HR efficiency was determined by FACS. Each bar represents the mean ± SD for triplicate experiments (upper, *P < 0.05). The knockdown efficiency of USP11, RNF20, BMI1 and BRCA1 was examined by western blotting (lower). (**C**) EJ5-HEK293 cells treated with USP11, and/or RNF20, BMI1, Ku80 siRNAs were transfected with I-SceI, and NHEJ efficiency was determined by FACS. Each bar represents the mean ± SD for triplicate experiments (upper, *P < 0.05). The knockdown efficiency of USP11, RNF20, BMI1 and Ku80 was examined by western blotting (lower). (**D**, **E**) DR-GFP-U2OS (D) or EJ5-GFP-HEK293 (E) cells were transfected with USP11 3′UTR siRNAs or/and HA-I-SceI, USP11 or USP11/C318S expression plasmids and collected for FACS analysis. Each bar represents the mean ± SD for triplicate experiments (*P < 0.05).

IRIF of BRCA1 and 53BP1 represents two major DSB repair pathways, HR and NHEJ, respectively (84–86). To further support USP11-catalyzed histone deubiquitination and to explore its functional significance in DNA damage response, U2OS cell clones with USP11 stably depleted were co-transfected with vector, USP11 or USP11/C318S plasmids. Immunofluorescent staining followed by high-content microscopy showed that USP11 depletion was associated with a mild nevertheless significant and reproducible increase in γH2AX, BRCA1 and 53BP1 IRIF during DNA damage repair (Figure 5A), indicative of a impaired repair efficiency in USP11-depleted cells. Meanwhile, wild-type USP11, but not USP11/C318S, was able to rescue the retention of DNA repair factors induced by USP11 depletion, suggesting that the impact of USP11 on repair factors recruitment is dependent on its deubiquitinase activity.

To further address the extent of USP11 in affecting DDR, GFP-based chromosomal reporter assays were used to measure HR or NHEJ repair efficiency in two stable cell lines DR-GFP-U2OS and EJ5-GFP-HEK293, respectively (45–47). The HR repair efficiency is manifested by the percentage of cells expressing GFP protein in U2OS cells transfected with endonuclease I-SceI. USP11 knockdown resulted in a decrease in the relative percentage of GFP-positive U2OS cells (Figure 5B), comparable to the effect of depletion of BRCA1, a key regulator of HR repair (84), and depletion of RNF20 or BMI1 restored the deficiency of HR repair in USP11-depleted DR-GFP-U2OS cells (Figure 5B). Analogously, depletion of USP11 in HEK293 cells stably integrated with an EJ5-I-SceI cassette led to a marked reduction of the percentage of GFP-positive cells (Figure 5C), similar to the effect of depletion of Ku80, an essential component of NHEJ repair (87), and RNF20 or BMI1 knockdown rescued the deficiency of NHEJ repair in USP11-depleted cells (Figure 5C). Moreover, depletion of RNF20 or BMI1 reversed the increase in γH2AX foci resolution in USP11-depleted cells (Supplementary Figure S5G), supporting a note that H2BK120 and H2AK119 deubiquitination are important for USP11-mediated DNA repair.

We then verified whether the deubiquitinase activity of USP11 is required for its involvement in DDR. To this end, DR-GFP-U2OS cells were co-transfected with the I-SceI plasmid and siRNA targeting 3′-UTR of USP11 together with USP11 or USP11/C318S. FACS analysis demonstrated that wild-type USP11, but not USP11/C318S, was able to rescue the decreased HR repair efficiency induced by depletion of endogenous USP11 (Figure 5D). Similarly, overexpression of USP11, but not USP11/C318S, offset the deficiency of NHEJ repair in USP11-depleted cells (Figure 5E). Collectively, these results suggest that USP11 is involved in a timely disassemble of DNA repair factors and in efficient HR and NHEJ repair dependent on its deubiquitinase activity.

### USP11 is required for chromatin condensation and genomic stability

**Figure 6.**
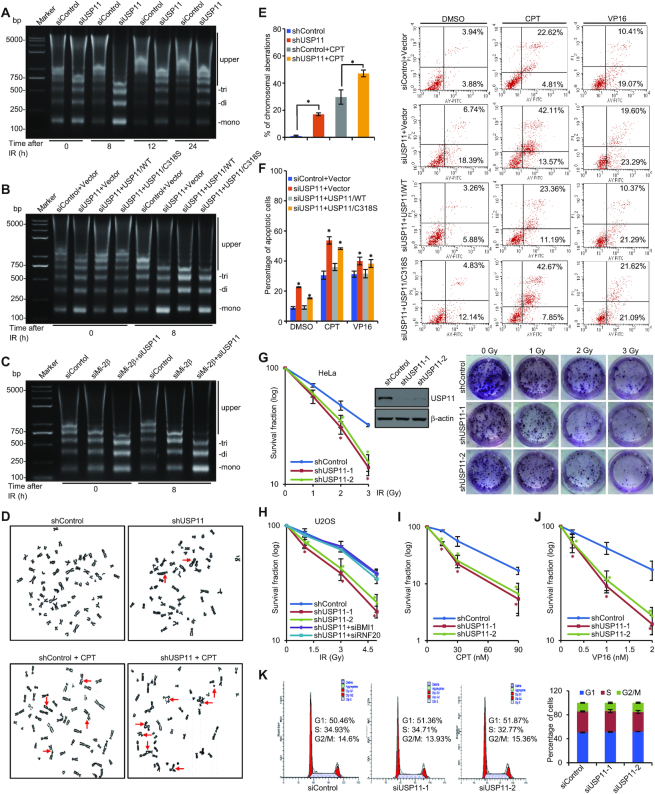
USP11 is Required for Chromatin Condensation and Genomic Stability. (**A**) U2OS cells were transfected with control or USP11 siRNAs, exposed to X-ray IR (10 Gy) and collected at different time points post IR. Nuclei were incubated with 2.5 U/ml MNase and purified and separated by electrophoresis in 1.2% agarose gel. Mono-, di-, tri- and upper nucleosome are indicated. (**B**) U2OS cells transfected with USP11 3′UTR siRNAs together with USP11 or USP11/C318S expression plasmids were exposed to X-ray IR (10 Gy) and subjected to MNase sensitivity assays at 8 h after irradiation. (**C**) U2OS cells treated with Mi-2β siRNAs and/or USP11 siRNAs were exposed to X-ray IR (10 Gy) and collected at 8 h post IR for MNase sensitivity assays. (**D**) U2OS clones with control or USP11 stably depleted were treated with DMSO or 2 μM CPT for 12 h, incubated in a final concentration of 1 μg/ml colcemid for 4 h followed by metaphase spreads preparation. *Arrows* indicate chromosome aberrations. (**E**) The percentage of chromosomal aberrations was quantified. Each bar represents the mean ± SD for triplicate experiments and at least 50 metaphases were counted in each experiment (*P < 0.05). (**F**) HeLa cells transfected with control or USP11 3′UTR siRNAs together with USP11 or USP11/C318S were treated with DMSO, CPT (1 μM, 48 h) or VP16 (40 nM, 48 h) and collected for annexin V and propidium iodide double staining. Cell apoptosis was determined by flow cytometry (right). Quantification was determined as mean ± SD for triplicate experiments (left, *P < 0.05). (**G**) HeLa clones with control or USP11 stably depleted were treated with X-ray IR at the indicated doses and subjected to clonogenic survival assays. Error bars represent mean ± SD for triplicate experiments (*P < 0.05). The representative images from biological triplicate experiments are shown. (**H**) U2OS clones with control or USP11 stably depleted were transfected with RNF20 or BMI1 siRNAs, and treated with IR at the indicated doses and subjected to clonogenic survival assays. Error bars represent mean ± SD for triplicate experiments (*P < 0.05). (**I, J**) U2OS clones with control or USP11 stably depleted were treated with CPT (I) or VP16 (J) at the indicated doses and subjected to clonogenic survival assays. Error bars represent mean ± SD for triplicate experiments (*P < 0.05). (**K**) U2OS cells were treated with control or USP11 siRNAs, and cell cycle profiles were analyzed by FACS.

Given the important role of USP11-catalyzed histone deubiquitination in DNA damage response, it is expected that USP11 is associated with ubiquitin signal clearance and subsequent chromatin restoration during the late stage of DNA repair (88). To test this, we performed micrococcal nuclease (MNase) sensitivity assays in USP11-depleted U2OS cells under the treatment of X-ray-generated IR. USP11 depletion led to an overt increase in MNase sensitivity of chromatin at all recovery time points after IR exposure, compared to control, and the difference was much evident at 8 h post IR (Figure 6A and Supplementary Figure S6A). Moreover, the role of USP11 in chromatin compaction is specific and through its catalytic activity, as wild-type USP11 overexpression in USP11-depleted U2OS cells was able to offset siUSP11-asscoiated increase in chromatin accessibility, whereas overexpression of USP11/C318S failed to do so (Figure 6B and Supplementary Figure S6B). Meanwhile, Mi-2β knockdown also resulted in an increase in chromatin accessibility, and simultaneous knockdown of Mi-2β and USP11 aggravated the situation (Figure 6C and Supplementary Figure S6C). These observations support the argument that USP11 is functionally involved in chromatin compaction during the late stage of DNA repair. Furthermore, metaphase analysis of Giemsa-stained chromosomes showed that knockdown of USP11 caused a remarkable increase in chromosomal aberrations, regardless with or without CPT treatment (Figure 6D and E), suggesting that USP11 is functionally involved in the maintenance of the genome stability.

To strengthen the functional significance of USP11-mediated histone deubiquitination, we tested the effect of USP11 on cell apoptosis and survival in response to DNA damaging agents. For this purpose, HeLa cells were co-transfected with USP11 3′UTR-siRNAs and USP11 or USP11/C318S and treated with CPT or VP16. Flow cytometry analysis showed that knockdown of USP11 promoted cell apoptosis, and CPT or VP16 treatment aggravated USP11 depletion-induced cell apoptosis (Figure 6F). The cell apoptosis-promoting effect under USP11 depletion was alleviated, to some extent, by simultaneous expression of wild-type USP11, but not USP11/C318S (Figure 6F), indicating that the role of USP11 in cell apoptosis is dependent on its enzymatic activity. In addition, we used a clonogenic survival assays to evaluate if USP11 plays any roles in cell survival following DNA damage. In these experiments, HeLa cells with USP11 stably depleted were exposed to different dosage of X-ray IR. USP11 deficiency was associated with a significantly compromised cell survival in response to IR (Figure 6G). Similar results were obtained in IR-treated U2OS cells, furthermore we found RNF20 or BMI1 depletion rescued the sensitivity to IR exposure in USP11-depleted cells, supporting that H2BK120 and H2AK119 deubiquitination are important for USP11-mediated cell survival (Figure 6H). Analogously, USP11 depletion resulted in an increase in sensitivity of U2OS cells to CPT or VP16 treatment (Figure 6I and J). Meanwhile, USP11 knockdown had no evident effects on cell cycle profiles, indicating that USP11-induced phenotypes in response to DNA damage are not due to the effect of USP11 on overall cell cycle progression (Figure 6K). U2OS clones with control or USP11 stably depleted were synchronized at G_1_/S boundary by double thymidine blocking and subsequently released into cell cycle. We found the expression of USP11 protein level, the catalytic activity for H2AK119ub and H2BK120ub, and the ability to interact with NuRD subunits are independent on cell cycle progression (Supplementary Figure S7A and B), suggesting the role of USP11 in histone deubiquitination is not cell-cycle regulated. These observations suggest that USP11 protects cells from genotoxic insults and promotes cell survival. Collectively, these findings support a notion that USP11 is an important chromatin modifier that acts to protect cells from genotoxic insults and contribute to chromatin condensation, genomic stability, and eventually cell survival.

## DISCUSSION

The DNA damage response constitutes a vast signaling network that temporarily modulates numerous aspects of cellular activities in the face of DNA lesions, especially the severe lesions such as DSBs (2). DSBs elicit a hierarchical process executed through a series of PTMs to modify the relevant proteins or the structure of the chromatin surrounding the break (6,7,18,29). PTMs are dynamically regulated, functionally coordinated, and constantly balanced between addition and removal to guide DDR. For example, ATM-dependent phosphorylation and subsequent E3 ligases-mediated ubiquitination play a critical role in damage checkpoint activation and timely initiation of repair by providing the recruitment platform for subsequent repair proteins (18,23), and it is reported that USP44 inhibits RNF8/RNF168-mediated H2AK15ub and subsequent 53BP1 retention at DSB sites (49), while PP2A, PP4C, PP6 and Wip1 catalyze γH2AX dephosphorylation during recovery from the DNA damage checkpoint (89–93). Tip60 is one of the first modifiers recruited to DNA break sites to acetylate H4 and H2A, leading to the relaxation of the chromatin (94,95). Through a systematic screening for DUBs that act in DSB responses, we found that USP11 is implicated in efficient DNA repair as a deubiquitinase for H2AK119ub and H2BK120ub. USP11 depletion leads to a significant increase and slower clearance in the level of H2AK119ub and H2BK120ub during DNA repair process after IR exposure or MMC treatment, and the delay of histone ubiquitination removal causes inefficient DNA damage repair and induces genomic instability. A previous study reported that USP11 deubiquitylates γH2AX both *in vivo* and *in vitro* but not H2AK119ub and H2BK120ub *in vitro* (43). Differences in preparation of the substrates and in purification of the enzyme in *in vitro* deubiquitylation experiments may contribute to the discrepancy of observations, and in fact there was a slight decrease in H2BK120ub in the previous study (43). We performed *in vitro* deubiquitination assays using bacterially expressed GST-USP11 protein, as USP11 purified from mammalian cells might be contaminated with other proteins such as E3 ubiquitin ligases. Our study suggests that USP11 acts as a *bona fide* histone deubiquitinase for H2AK119ub and H2BK120ub, and we propose that USP11 is required for efficient HR and NHEJ repair in response to DNA damage and for proper dissociation of DNA repair proteins.

The sensing and repair of DNA damages occur not only at the DNA level, but also in chromatin context in eukaryotic cells since DNA is protected by higher-order chromatin fibers. The chromatin remodeling is required to confront this physical barrier to enzymes and regulatory factors to access to the site of DNA-damaged and subsequent chromatin recovery during the late stage of DDR (96). Given the intimate connection of chromatin remodeling to DNA damage repair, it is logical to believe that chromatin remodeling plays a central rolein DDR. Indeed, it has been reported the both human ISWI ATPases SMARCA5 and SMARCA1 are rapidly recruited to DSBs and their knockdown renders cells hypersensitive to DNA damage (97). Chromatin-remodeling factor Mi-2β, ATPases subunit of the human NuRD complex, facilitates both checkpoint signaling and repair events after DNA damage (71). Moreover, the *erg-1* gene (the *C. elegans* ortholog of mammalian MTA2) is one of the candidate genes that protect animal cells against IR by a genome-wide RNAi screening (98). Our current findings that USP11 is enriched in DNA damage sites and is physically associated with and functional linked to NuRD complex are consistent with these previous observations.

We noticed the levels of H2AK119ub, H2BK120ub and H3ac peaked at 1∼4 h after IR exposure and gradually decreased thereafter till 12 h as showed in Figure 4A, consistent with the observation that USP11 recruitment in DNA damage sites peaked at 4 h post-IR (Figure 3G). Considering the observation that USP11 knockdown led to an increased retention of DNA repair factors after IR exposure (Figure 5A), our study suggests that USP11 influences DNA repair process at the middle and late periods of DNA damage response to ensure proper disassembly of DNA repair factors and chromatin reorganization. Given that drugs targeting chromatin-modifying enzymes are being explored as anticancer therapies, both alone and in combination with DNA-damaging treatments, defining the role of the USP11/NuRD complex in DDR functions will aid the effort in developing promising cancer therapeutics.

Multiple lines of evidence indicate that NuRD complex associates with transcription factors to promote transcriptional repression of downstream targets. It has been reported that NuRD complex enrichment at DNA-damage sites brings a transiently and locally repressive chromatin structure to block transcription and facilitate DNA-damage signaling and/or repair (65). Consistently, it has been shown that the bromodomain protein ZMYND8 recruits NuRD complex to transcription-associated DNA damage sites and promotes homologous recombination (99). Further studies showed that ZMYND8 co-localizes with NuRD on target genes and regulates poly(ADP-ribose)-dependent recruitment of GATAD2A/NuRD to damaged chromatin (100). In addition, a proteomics study that coupled isotopic labeling with chromatin fractionation identified three members of the polycomb complex, EZH2, CBX8 and SUZ12, are recruited by PARP to DNA lesions following UV laser microirradiation (74). Indeed, we found that USP11 is enriched in damaged chromatin in a PARP1-dependent manner, supporting the physical association and functional connection between USP11 and the NuRD complex in response to DNA damage. Given the deubiquitinase activity of USP11 toward H2BK120ub, the mark necessary for stimulation of the Pol II elongation (101), it is tempting to speculate that USP11 similarly modulates chromatin structure locally at sites of DNA breaks and contributes to transient transcription repression to facilitate DNA repair. Consistently, endonuclease AsiSI assays followed by qChIP analysis (Figure 4D and Supplementary Figure S5B–E) indicated that acetylation and ubiquitination of histone crosstalk through a synergistic effect of the USP11/NuRD complex occur specifically around DNA break regions in cellular response to DNA damage. Furthermore, the immunostaining assays with H2BK120ub and FK2 antibodies showed in microirradiated cells USP11 knockdown led to increases in immunofluorescent intensity of H2BK120ub and conjugated ubiquitin not only in laser-inflicted DNA damage tracks but also in non-damaged sites of nucleus (Supplementary Figure S5F), together with the observation in Figures 1A, 2F, 4A–C, suggesting USP11 mediated-histone deubiquitination is not merely confined to the DSB regions and is independent of DNA damage. Our study provides an example of how DDR takes full advantage of functional complex(s) to regulate its process, and evidence for the functional interplay between DDR, gene transcription, and chromatin organization.

While we interpret the observed USP11 knockdown phenotypes stemmed from USP11-catalyzed histone deubiquitination, we do not exclude the possibility that additional targets for USP11 exist and contribute to the phenotype. Consistently, depletion of RNF20 or BMI1 restored the deficiency of DNA repair and clonogenic survival assays in USP11-depleted cells, indicative that deubiquitination of H2BK120 and H2AK119 is not all, but at least important for USP11-mediated DNA repair. In addition, in light of histone crosstalks between H2AK119ub and H3K27me3, H2BK120ub and H3K4me3 or H3K79me2 (102), questions remain as to whether the recruitment of ‘writer’, ‘eraser’ or ‘reader’ of these histone marks such as PRC2 is affected by USP11 knockdown and contributes to the phenotypes we observed in USP11-depleted cells. We also found that the exposure of IR or DNA-damaging agents enhanced protein level of USP11 and affected the formation of the USP11/NuRD complex, consistent with the previous reports (38,40). However, it has been reported that there is a loss of USP11 steady-state level in G_1_ phase upon 20 Gy IR treatment (41), so we guessed that exposure to different dose of IR and cell-cycle specificity may be partly responsible for the discrepancy of our observation. Thus how USP11 is regulated at transcriptional or/and post-translational level in responding to DNA damages and cell cycle progression needs to be further studied. Moreover, the extraordinary level of substrate diversity and the magnitude of USP11 involvement in NER, NHEJ and HR process define USP11 as a multifaceted player and faithful keeper of genomic integrity (40,41,44). It will also be interesting to investigate the mechanistic basis underlying USP11-dedicated DSB repair pathways and the relationship between substrate diversity and the distinct cellular activities of USP11. In addition, spatiotemporal resolution of the recruitment to and extraction from DNA damage sites of the USP11/NuRD complex, the fluctuation of histone PTMs levels and consequent chromatin reorganization will surely add to the understanding of not only the functional dynamics of the USP11/NuRD complex, but also the insight into the DNA repair process. Furthermore, the role of the USP11/NuRD complex in gene transcription and other cellular processes will be an interesting topic in the future investigations. Nevertheless, the extraordinary level of substrate diversity and the magnitude of USP11 involvement in NER, NHEJ and HR suggests USP11 is a multifaceted regulator and faithful keeper of genome integrity.

## Supplementary Material

gkz726_Supplemental_File

## Notes

Present address: Luyang Sun, Department of Biochemistry and Molecular Biology, School of Basic Medical Sciences, Peking University Health Science Center, 38 Xueyuan Road, Beijing 100191, China.
