# Supplementary material for: USP11 acts as a histone deubiquitinase functioning in chromatin reorganization during DNA repair
Source: Nucleic Acids Res. 2019 Aug 28;47(18):9721–40. doi: 10.1093/nar/gkz726 (PMC6765148; doi:10.1093/nar/gkz726)
Supplement: gkz726_Supplemental_File [file gkz726_supplemental_file.pdf]

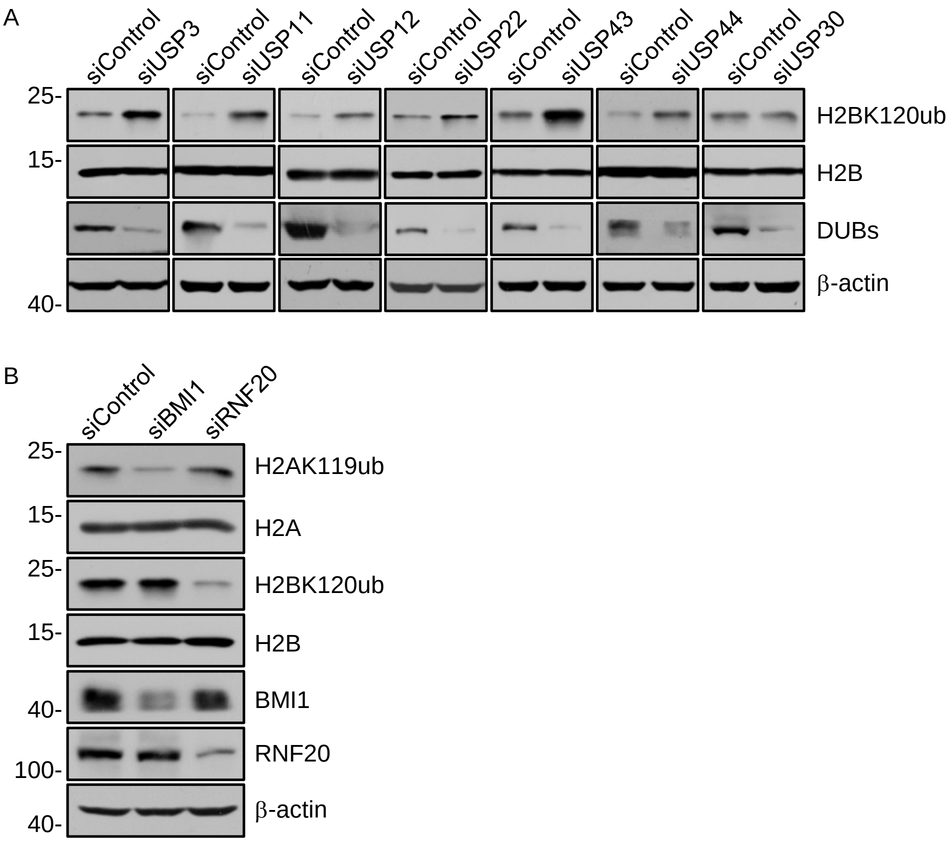

Figure S1. Screening for DUBs that Affect H2BK120 Ubiquitination. (A) The level of the indicated histone marks or proteins was measured by western blotting in HeLa cells transfected with siRNAs against USP3, USP11, USP12, USP22, USP43, USP44 or USP30. (B) Western blotting analysis of the indicated histone marks or proteins in HEK293T cells treated with control, BMI1, or RNF20 siRNAs.

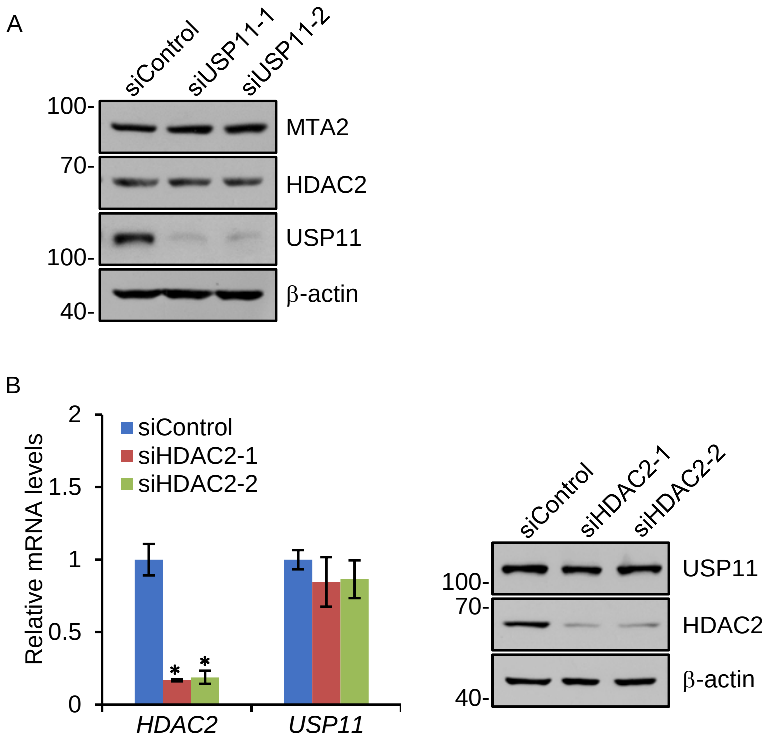

Figure S2. USP11 Does not Regulate the Protein Stability of NuRD Components and HDAC2 Does not Affect the Expression of *USP11*. (A) Western blotting analysis of the indicated protein expression in HEK293T cells treated with control or USP11 siRNAs. (B) qPCR measurement of mRNA expression of HDAC2, USP11 in HEK293T cells upon HDAC2 depletion. Error bars represent mean ± SD for triplicate experiments ($*p < 0.05$). Western blotting analysis of the indicated protein expression in HEK293T cells treated with control or HDAC2 siRNAs.

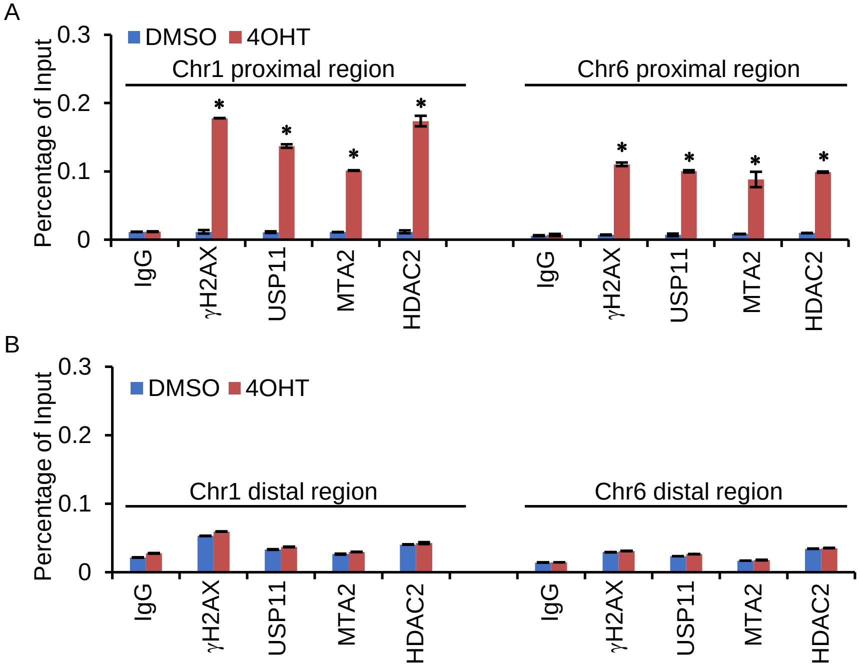

Figure S3. The Recruitment of USP11 and NuRD in DSB sites by a DIvA System. (A, B) U2OS cells stably expressing HA-AsiSI were treated with DMSO or 1 μM 4OHT for 4 h. qChIP experiments were performed using indicated antibodies with primers that cover the DNA sequences flanking the AsiSI site and the break distal region in Chr1 or Chr6. Each bar represents the mean ± SD for triplicate experiments (*$p < 0.05$).

Figure S4

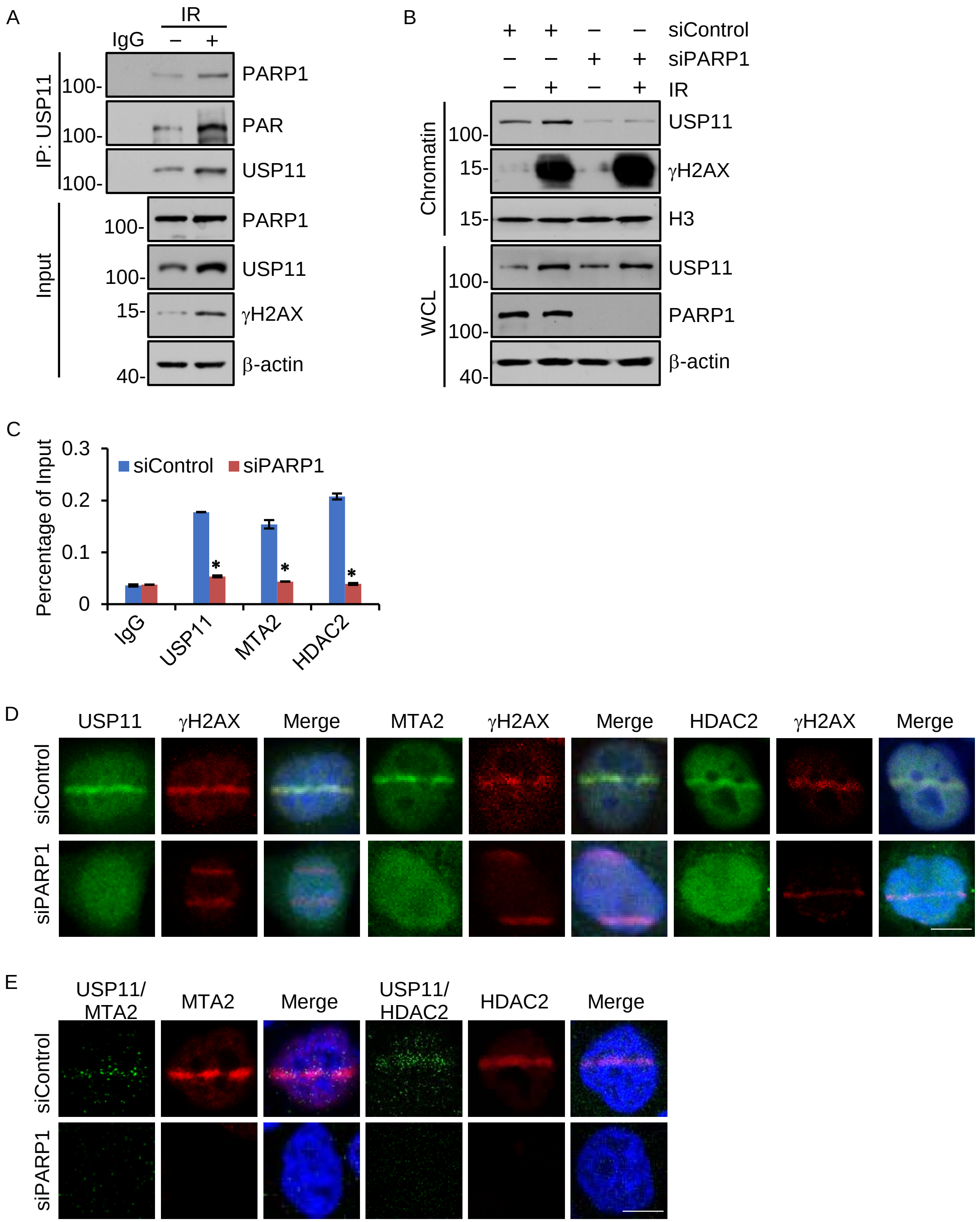
A
IR
IgG – +
IP: USP11
100-
PARP1
100-
PAR
100-
USP11
Input
100-
PARP1
100-
USP11
15-
γH2AX
40-
β-actin
B
+ + – – siControl
– – + + siPARP1
– + – + IR
Chromatin
100-
USP11
15-
γH2AX
15-
H3
WCL
100-
USP11
100-
PARP1
40-
β-actin
C
Percentage of Input
0.3
0.2
0.1
0
siControl
siPARP1
IgG
USP11
MTA2
HDAC2
D
USP11
γH2AX
Merge
MTA2
γH2AX
Merge
HDAC2
γH2AX
Merge
siControl
siPARP1
E
USP11/ MTA2
MTA2
Merge
USP11/ HDAC2
HDAC2
Merge
siControl
siPARP1

Figure S4. PARP1 Recruits USP11/NuRD to DNA Lesions. (A) U2OS cells were collected at 8 hour post-IR (6 Gy), cellular extracts were immunoprecipitated and then immunoblotted with antibodies as indicated. (B) U2OS cells pre-treated with PARP1 siRNAs were exposed to X-ray IR (6 Gy) and collected at 1 hour post-IR. Whole-cell lysates and chromatin fractions were extracted for western blotting analysis with the indicated antibodies. (C) U2OS cells stably expressing HA-AsiSI were treated with PARP1 siRNAs in presence of 1 μM 4OHT for 4 h. qChIP experiments were performed using antibodies as indicated with primers that cover the DNA sequences flanking the AsiSI site in Chr22. Each bar represents the mean ± SD for triplicate experiments (*p < 0.05). (D) U2OS cells were subjected to a ultraviolet-A laser (λ=355 nm, 40% energy) with PARP1 siRNAs pre-treated and immunofluorescent analysis with the indicated antibodies at 4 h after micro-IR were performed. γH2AX was used as a positive control. Bar, 5 μm. (E) U2OS cells were subjected to a ultraviolet-A laser (λ=355 nm, 40% energy) with PARP1 siRNAs pre-treated and were collected at 4 h after micro-IR. Duolink PLA was performed with anti-USP11 and anti-MTA2 or anti-USP11 and anti-HDAC2 antibodies. PLA signals were shown in green, staining of MTA2 or HDAC2 was shown in red, and nucleic were stained blue by DAPI. Bar, 5 μm.

Figure S5

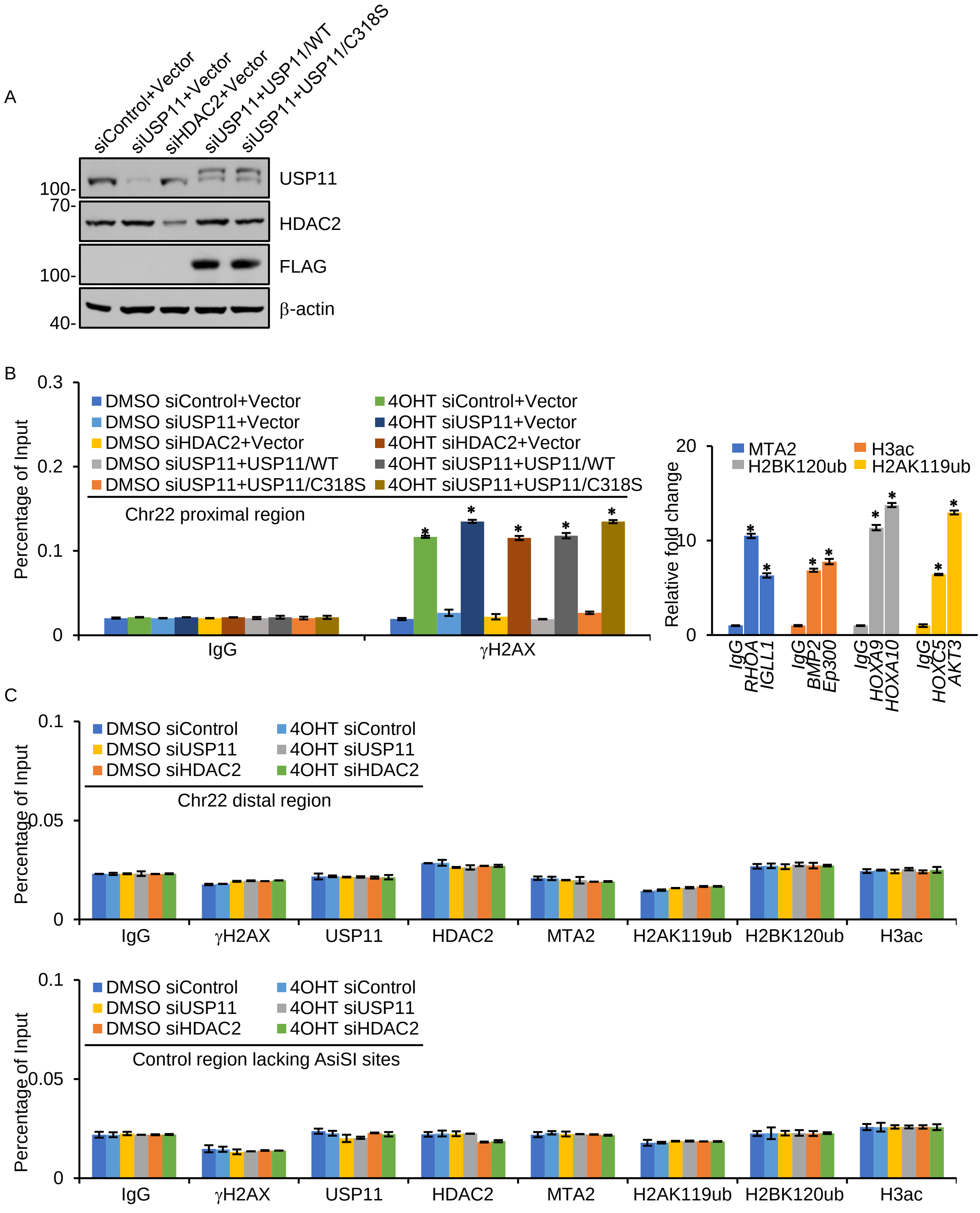

A
siControl+Vector
siUSP11+Vector
siHDAC2+Vector
siUSP11+USP11/WT
siUSP11+USP11/C318S
USP11
HDAC2
FLAG
β-actin
100-
70-
100-
40-
B
Percentage of Input
DMSO siControl+Vector
DMSO siUSP11+Vector
DMSO siHDAC2+Vector
DMSO siUSP11+USP11/WT
DMSO siUSP11+USP11/C318S
4OHT siControl+Vector
4OHT siUSP11+Vector
4OHT siHDAC2+Vector
4OHT siUSP11+USP11/WT
4OHT siUSP11+USP11/C318S
Chr22 proximal region
IgG
γH2AX
Relative fold change
MTA2
H3ac
H2BK120ub
H2AK119ub
IgG
RHOA
IGLL1
IgG
BMP2
Ep300
IgG
HOXA9
HOXA10
IgG
HOXC5
AKT3
C
Percentage of Input
DMSO siControl
DMSO siUSP11
DMSO siHDAC2
4OHT siControl
4OHT siUSP11
4OHT siHDAC2
Chr22 distal region
IgG
γH2AX
USP11
HDAC2
MTA2
H2AK119ub
H2BK120ub
H3ac
Control region lacking AsiSI sites

Figure S5

D

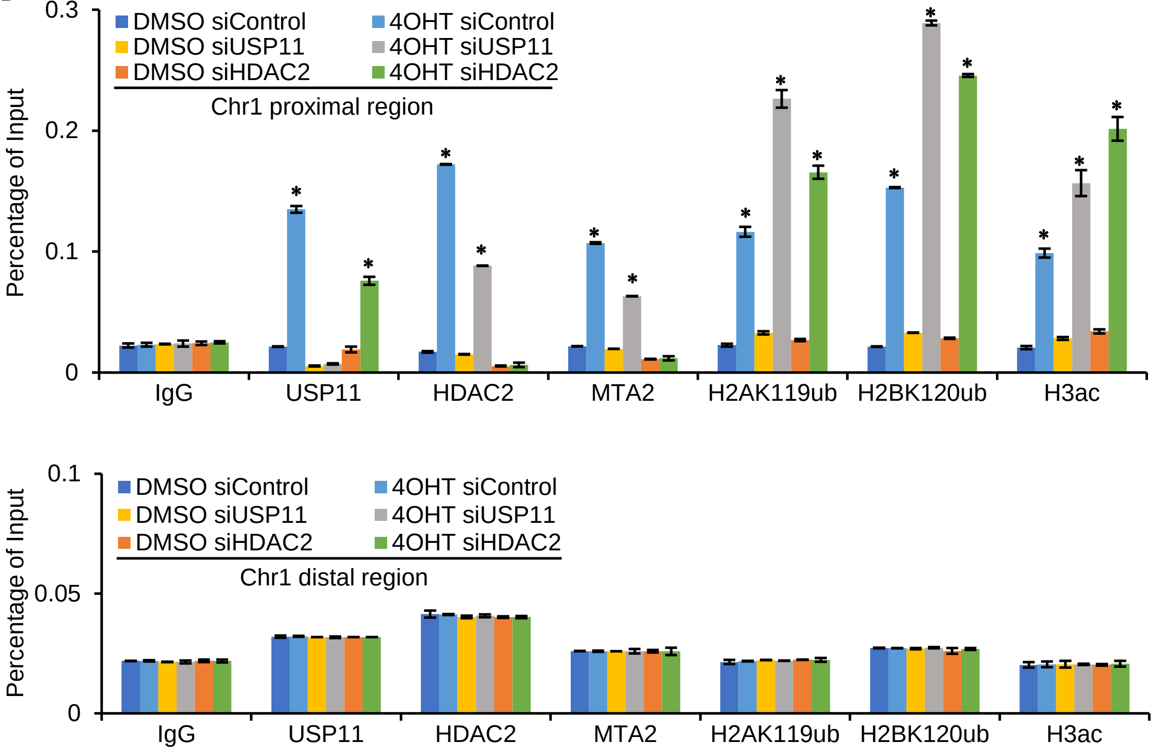

Percentage of Input
0.3
0.2
0.1
0
DMSO siControl
DMSO siUSP11
DMSO siHDAC2
4OHT siControl
4OHT siUSP11
4OHT siHDAC2
Chr1 proximal region
IgG
USP11
HDAC2
MTA2
H2AK119ub
H2BK120ub
H3ac
0.1
0.05
Chr1 distal region

E

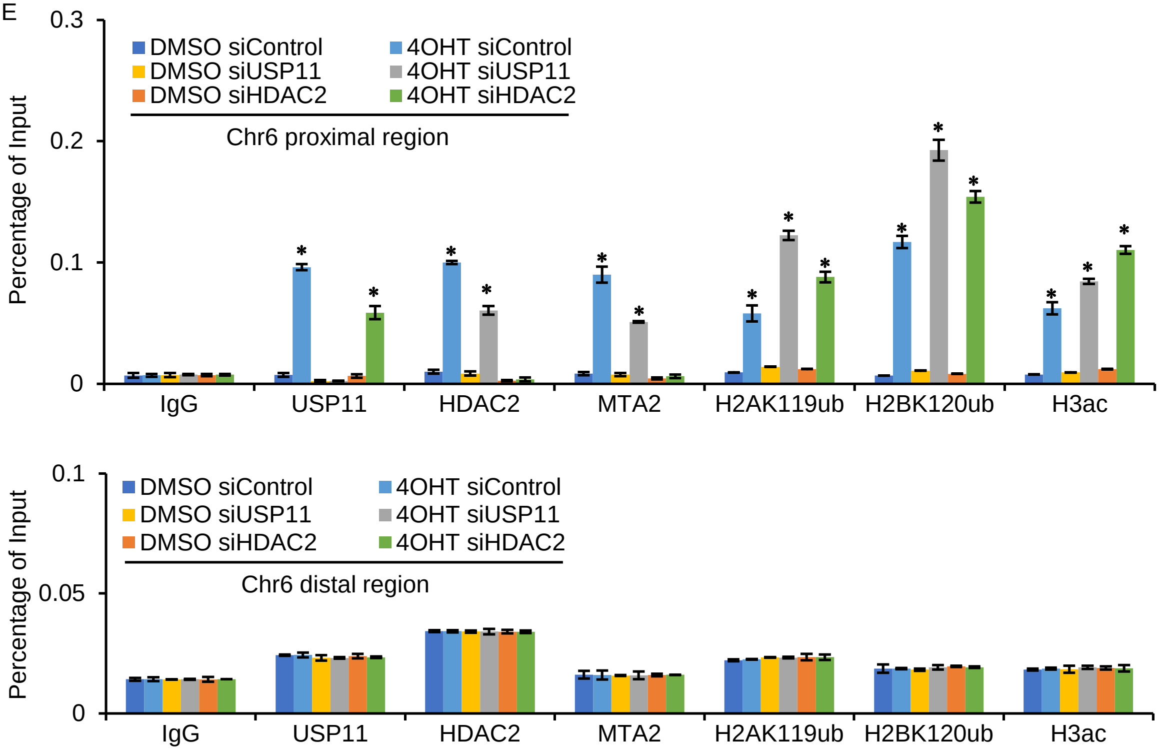

Percentage of Input
0.3
0.2
0.1
0
DMSO siControl
DMSO siUSP11
DMSO siHDAC2
4OHT siControl
4OHT siUSP11
4OHT siHDAC2
Chr6 proximal region
IgG
USP11
HDAC2
MTA2
H2AK119ub
H2BK120ub
H3ac
0.1
0.05
Chr6 distal region

Figure S5

F

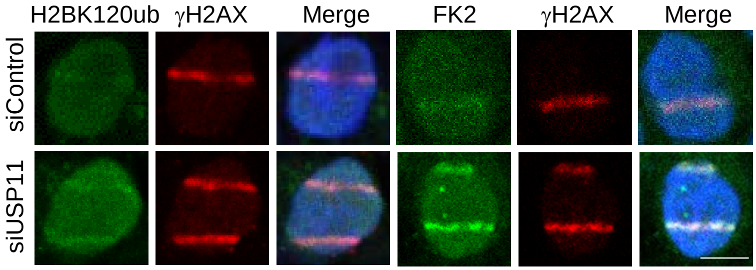

H2BK120ub
γH2AX
Merge
FK2
γH2AX
Merge
siControl
siUSP11

G

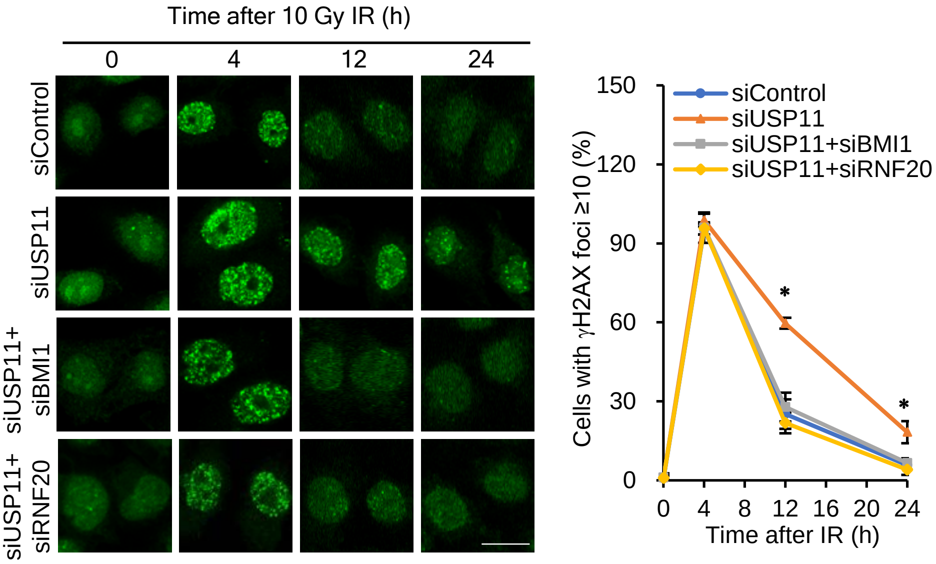

Time after 10 Gy IR (h)
0
4
12
24
siControl
siUSP11
siUSP11+ siBMI1
siUSP11+ siRNF20
siControl
siUSP11
siUSP11+siBMI1
siUSP11+siRNF20
Cells with γH2AX foci ≥10 (%)
150
120
90
60
30
0
*
*
0
4
8
12
16
20
24
Time after IR (h)

Figure S5. Crosstalk between Histone Deubiquitination and Deacetylation in DNA Damage Response. (A) U2OS cells stably expressing HA-AsiSI were cotransfected with siRNAs targeting USP11 3'-UTR, or HDAC2, and/or wild-type USP11 or USP11/C318S expression plasmids in absence or presence of 1 μM 4OHT. The expression level of proteins was monitored by western blotting analysis with the indicated antibodies. (B) U2OS cells stably expressing HA-AsiSI were treated with DMSO or 1 μM 4OHT for 4 h. qChIP experiments were performed using γH2AX antibodies with primers that cover the DNA sequences flanking the AsiSI site in Chr22 (left), or using antibodies against MTA2, H3ac, H2BK120ub, or H2AK119ub with primers that cover the promoter of indicated genes in U2OS cells (right). Each bar represents the mean ± SD for triplicate experiments (*$p < 0.05$). (C) U2OS cells stably expressing HA-AsiSI were cotransfected with siRNAs targeting USP11 3'-UTR, or HDAC2, and/or wild-type USP11 or USP11/C318S expression plasmids treated with DMSO or 1 μM 4OHT for 4 h. qChIP experiments were performed using indicated antibodies with primers that cover the break distal region in Chr22 (upper) or control genomic sequence lacking AsiSI sites (lower). Each bar represents the mean ± SD for triplicate experiments (*$p < 0.05$). (D, E) U2OS cells stably expressing HA-AsiSI were pre-treated with siRNAs against USP11 or HDAC2 in absence or presence of 1 μM 4OHT. qChIP experiments were performed using indicated antibodies with primers that cover the DNA sequences flanking the AsiSI site (upper) and the break distal region (lower) in Chr1 or Chr6. Each bar represents the mean ± SD for triplicate experiments (*$p < 0.05$). (F) U2OS cells with USP11 siRNAs pre-treated were subjected to a ultraviolet-A laser (λ=355 nm, 40% energy) and immunofluorescent analysis with the indicated antibodies at 4 h after micro-IR were performed. γH2AX was used as a positive control. Bar, 5 μm. (G) U2OS cells were treated with control, USP11, or/and BMI1 or RNF20 siRNAs, exposed to X-ray IR (10 Gy) and subjected to immunofluorescent staining of γH2AX at different time points post-IR. High-content system with automatic image processing was applied to determine the number of IRIF per cell. More than 500 cells were analyzed in each group. Bar, 10 μm. The percentage of cells with γH2AX IRIF ≥10 per cell was quantified. Each bar represents the mean ± SD for triplicate experiments (*$p < 0.05$).

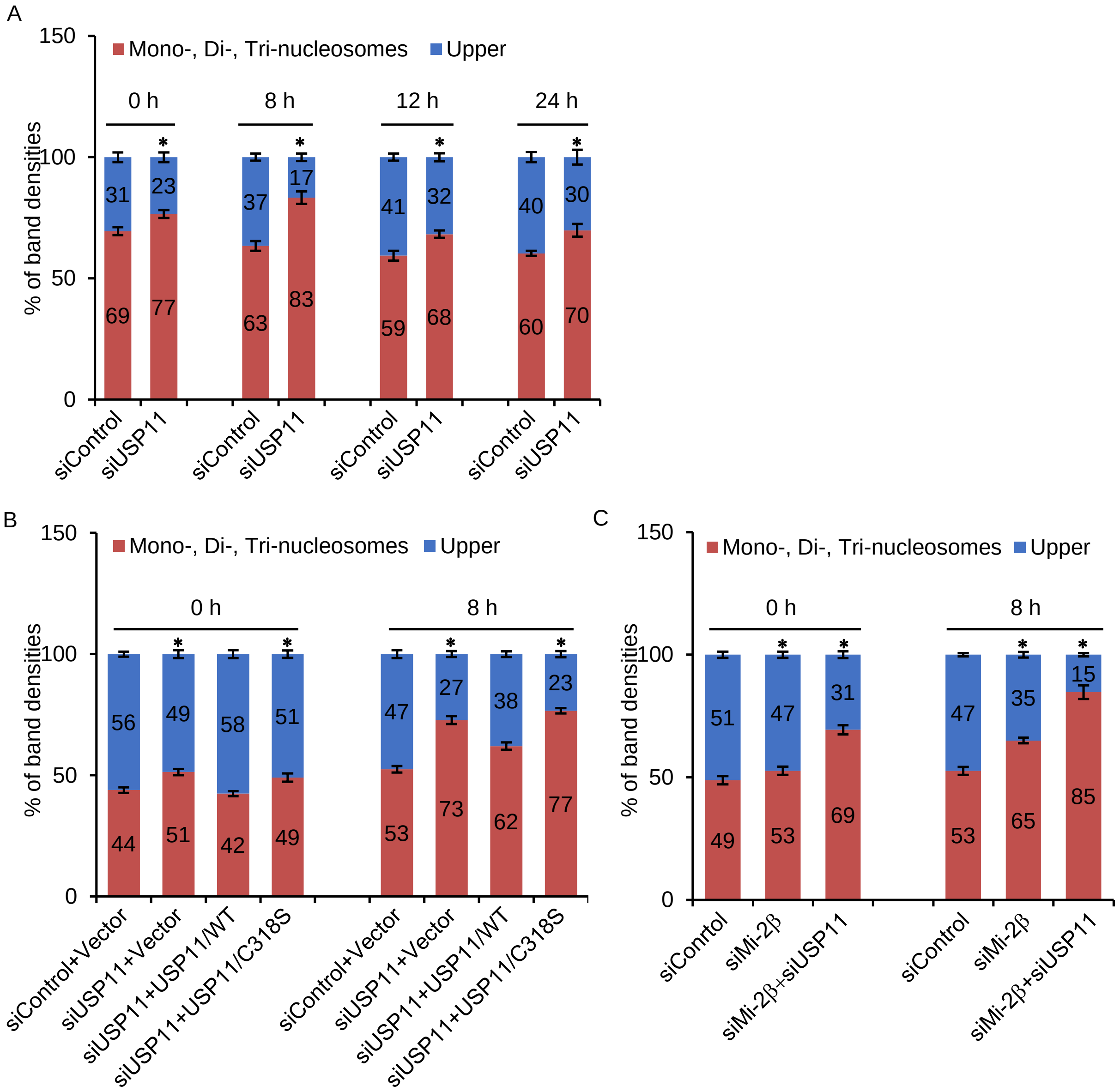

Figure S6. USP11 is Required for Chromatin Condensation. (A-C) The band densities of mononucleosome, dinucleosome, trinucleosome and higher molecular weight species (upper) in Figure 6A-C were quantified using ImageJ software and the intensity values were background subtracted. Error bars represent mean ± SD for triplicate experiments (**p* < 0.05).

Figure S7

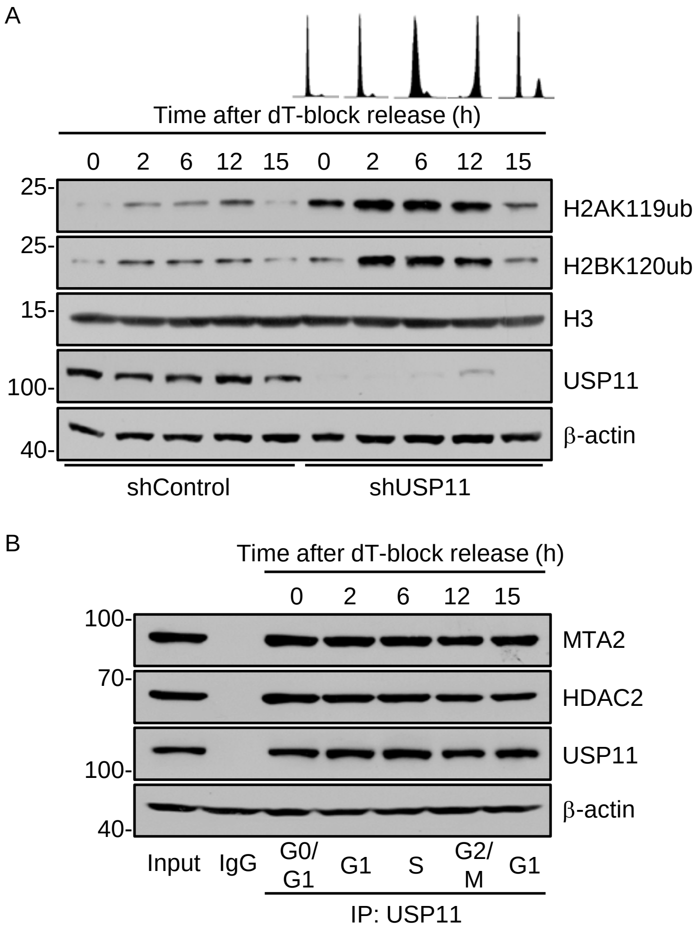

Figure S7. USP11 Protein Expression, the Catalytic Activity, and the Ability to Interact with NuRD Subunits Are Independent on Cell Cycle Progression. (A) U2OS clones with control or USP11 stably depleted were synchronized at G1/S border by double thymidine blocking, and were subsequently allowed to progress through the cell cycle for the indicated times monitored by flow cytometry (upper). The level of the indicated histone marks or proteins was analyzed by western blotting (lower). (B) U2OS cells were synchronized at G1/S border by double thymidine blocking, and were subsequently allowed to release for the indicated times. Cellular extracts were immunoprecipitated and then immunoblotted with antibodies as indicated.

Supplemental Table S1. Summary of Screen for 81 DUBs that Affect H2BK120 Ubiquitination or 53BP1 IRIF Formation.

Gene Symbol	H2BK120ub Decreased	Known Deubiquitinase for H2BK120	53BP1 IRIF Changed	Known Effect on 53BP1 IRIF	Known Participation in DSB repair
BAP1	–	NA	–	–	+
BRCC36	–	NA	+	+	+
COPS5	–	NA	–	NA	–
CYLD	–	NA	–	NA	–
DUB1A	–	NA	–	NA	–
DUB3	–	NA	+	+	+
JOSD1	–	NA	–	NA	–
MJD	–	NA	–	NA	–
MYSM1	–	NA	–	NA	–
OTUB1	–	NA	–	+	+
OTUB2	–	NA	–	+	+
OTUD1	–	NA	–	NA	–
OTUD4	–	NA	–	NA	–
OTUD5	–	NA	–	+	+
OTUD6B	–	NA	–	NA	–
OTUD7A	–	NA	–	NA	–
OTUD7B	–	NA	–	NA	–
PRPF8	–	NA	–	–	+
PSMD14	–	NA	–	–	+
STAMBP	–	NA	–	NA	–
UCHL1	–	NA	–	NA	–
UCHL3	–	NA	–	–	+
UCHL5	–	NA	–	–	+
USP1	–	NA	–	+	+
USP2	–	NA	–	NA	–
USP3	+	+	–	+	+
USP4	–	NA	–	–	+
USP5	–	NA	–	–	+
USP6	–	NA	–	NA	–
USP7	–	+	–	+	+
USP8	–	NA	–	–	+
USP9X	–	NA	–	NA	–
USP9Y	–	NA	–	–	–
USP10	–	–	–	–	–
USP11	+	NA	+	+	+
USP12	+	+	+	–	–
USP13	–	NA	–	–	+
USP14	–	NA	+	+	+
USP15	–	+	+	–	–

USP16	–	–	–	+	+
USP17	–	NA	–	–	–
USP18	–	NA	–	–	–
USP19	–	NA	–	–	+
USP20	–	NA	–	–	+
USP21	–	NA	–	–	+
USP22	+	+	–	–	+
USP24	–	NA	–	NA	–
USP25	–	NA	–	–	–
USP26	–	NA	–	+	+
USP27X	–	+	–	–	–
USP28	–	–	–	+	+
USP29	–	+	–	+	+
USP30	–	NA	–	NA	–
USP31	–	NA	–	NA	–
USP32	–	–	–	NA	–
USP33	–	NA	–	NA	–
USP34	–	NA	–	+	+
USP35	–	NA	–	NA	–
USP36	–	+	–	NA	–
USP37	–	NA	+	+	+
USP38	–	NA	–	NA	–
USP39	–	NA	–	NA	–
USP40	–	NA	–	NA	–
USP41	–	NA	–	NA	–
USP42	–	+	–	NA	–
USP43	+	+	–	NA	–
USP44	+	+	–	+	+
USP45	–	NA	–	NA	–
USP46	–	+	–	NA	–
USP47	–	NA	–	NA	–
USP48	–	NA	–	NA	–
USP49	–	+	–	NA	–
USP50	–	NA	–	NA	–
USP51	–	NA	–	+	+
USP52	–	NA	–	NA	–
USP53	–	NA	–	NA	–
USP54	–	NA	–	NA	–
VCPIP1	–	NA	–	NA	–
YOD1	–	NA	–	NA	–
ZA20D1	–	NA	–	NA	–
ZRANB1	–	NA	–	NA	–

NA, Not analyzed

Supplemental Table S2. Mass Spectrometry Analysis of USP11-containing Proteins

MW	Identified proteins	Peptides
110 kDa	USP11	VQVEYKGETK
		IINEPTAAAIAYGLDKK
		TTPSYVAFTDTERLIGDAAK
		STAGDTHLGGEDFDNR
		ITITNDKGR
		VEIIANDQGNR
		TTPSYVAFTDTER
		ENKITITNDKGR
		IINEPTAAAIAYGLDK
		VEIIANDQGNRTTPSYVAFTDTER
		IQKLLQDFFNGKELNK
		ARFEELNADLFR
		LLQDFFNGKELNK
		STGKENKITITNDK
		ALRDAKLDK
		FEELNADLFR
		LIGDAAK
		LLQDFFNGK
		EIAEAYLGGK
		LRTAcER
		NTTIPTK
		AMTKDNNLLGK
		RNTTIPTK
75 kDa	MTA2	YQAEIPDRLVEGESDNRNQQK
		TLLADQGEIR
		TPTQLEGATR
		QFESLPATHIR
		AVGTFAR
		NGYDLAK
		EFEEESKQPGVSEQQR
		QLEDGRTLSDYNIQKESTLHLVLR

		TLSDYNIQK
		PSDTIENVK
		INALTAASEAAcLIVSVDETIKNPR
		SLERAEAGDNLGALVR
		AEAGDNLGALVR
70 kDa	HSP71	IQDKEGIPPDQQR
		AKIQDKEGIPPDQQR
		TLSDYNIQKESTLHLVLR
		ESTLHLVLR
		QLEDGRTLSDYNIQK
		LIFAGKQLEDGR
		IINEPTAAAIAYGLDKKVGAER
		IINEPTAAAIAYGLDKK
		TVTNAVVTVPAYFNDSQRQATK
70 kDa	HSP72	YQAEIPDRLVEGESDNRNQQK
		TLLADQGEIR
		TPTQLEGATR
		QFESLPATHIR
		AVGTFAR
		NGYDLAK
		EFEEESKQPGVSEQQR
55 kDa	HDAC2	DGIDDESYGQIFKPIISK
		YGEYFPGTGDLR
		IGPILDNSTLQSEVKPILEK
		TSAcGLFSVcYPR
		LAGGDWFTSR
		QAAEDKSWR
		YmVADKFTELQK
		DKAVESLR
48 kDa	RbAp48 (RBBP4)	GLGLDDALEPR
		VYSLFLDESR

		TEALTQAFRR
		GFFDPNTEENLTYLQLK
		AQQIHSQTSQQYPLYDLDLGK
		LNDSILQATEQR
		QLQNIIQATSR
46 kDa	RbAp46 (RBBP7)	AAAQYYLGNFR
		SALNDVTAAR
		LSLDGQGR
		TQPYDVYDQVEFDVPVGSR
		LDELEELLTNNR
		APGFAHLAGLDK
		GEFGVYLVSDGSSRPYR
